# Chitosan Nanoparticle-Mediated Delivery of *Alstonia venenata* R.Br. Root Methanolic Extract: A Promising Strategy for Breast Cancer Therapy in DMBA-Induced Breast Cancer in Sprague Dawley Rats

**DOI:** 10.3390/antiox13121513

**Published:** 2024-12-11

**Authors:** Aarthi Jeganathan, Karuppusamy Arunachalam, Anju Byju, Anju Rani George, Sradha Sajeev, Kavimani Thangasamy, Geetha Natesan

**Affiliations:** 1 Department of Botany, Bharathiar University, Coimbatore 641046, TN, India; aarthij96@gmail.com (A.J.); anjubyju2014@gmail.com (A.B.); anju.rani.nx@gmail.com (A.R.G.); sajeevsradha@gmail.com (S.S.); kavieswari007@gmail.com (K.T.); 2 Center for Studies in Stem Cells, Cellular Therapy and Toxicological Genetics (CeTroGen), Faculty of Medicine (FAMED), Federal University of Mato Grosso do Sul (UFMS), Campo Grande 79070-900, MS, Brazil; arunachalam04@gmail.com

**Keywords:** *Alstonia venenata*, chitosan nanoparticles, rats, dimethylbenz(a)anthracene, antioxidant activity, anticancer activity

## Abstract

*Alstonia venenata* R.Br., a plant native to the Western Ghats, is recognized for its diverse medicinal properties. The plant’s extracts, particularly rich in alkaloids and other bioactive compounds, have shown potential anticancer activity. This study investigates the therapeutic potential of chitosan nanoparticles (CNPs) loaded with the root methanolic extract (RME) of *A. venenata* in combating breast cancer induced by dimethylbenz(a)anthracene (DMBA) in female Sprague Dawley rats. The RME-loaded chitosan nanoparticles (RME-EnCNPs) were synthesized and characterized, and their in vivo efficacy was evaluated. Treatment with RME-EnCNPs significantly inhibited tumor progression, which is evidenced by reduced tumor volume, burden, and incidence. Moreover, the nanoparticles demonstrated a sustained release of the active compounds, leading to marked improvements in various biochemical, enzymatic, and histopathological parameters. The study found that both RME and RME-EnCNPs effectively suppressed tumor growth, with RME-EnCNPs showing superior efficacy in modulating tumor progression. Antioxidant assays revealed that treatment with RME-EnCNPs (500 mg/kg) resulted in significant increases in total protein, superoxide dismutase (SOD), catalase, glutathione peroxidase (GPx), and glutathione (GSH) levels, alongside a marked reduction in lipid peroxidation (LPO) (*p* < 0.001). These findings suggest that RME-EnCNPs exert a potent antioxidant effect, mitigating oxidative stress within the tumor microenvironment. The root extract of *A. venenata* and its nanoparticle formulation hold promise as a potential therapeutic agent for breast cancer, warranting further investigation to isolate active bioactive compounds and elucidate their mechanisms of action.

## 1. Introduction

Cancer, a multifaceted spectrum of diseases, is characterized by the unchecked proliferation of abnormal cells with the potential to spread to different parts of the body—a process known as metastasis. According to the World Health Organization (WHO) in 2022, metastases were identified as the primary cause of cancer-related fatalities, contributing to approximately 10 million deaths in 2020 [1]. Among the various forms of cancer, breast cancer stands out as the most prevalent malignancy in women, exhibiting considerable molecular heterogeneity. In 2022, there were 2.3 million new breast cancer diagnoses in women worldwide, and 670,000 women died from the disease. Breast cancer is the most prevalent cancer among Indian women, making up 28.2% of all cancer cases in the country [2]. Projections indicate a potential surge to 3.2 million new cases by 2040, underscoring the urgency for comprehensive research and therapeutic interventions [2]. The initiation of breast cancer in laboratory rodents, particularly Sprague Dawley and Wistar rats, often involves exposure to chemical carcinogens like DMBA and N-methyl nitrosourea [3]. Understanding the susceptibility of these rodents to carcinogens provides insights into the complex etiology of breast cancer. The development of chemopreventive medications has been instrumental in interrupting the carcinogenic cascade. These medications employ various mechanisms, including the enhancement of detoxifying enzymes, scavenging reactive oxygen species, and regulating apoptosis [4]. Despite significant therapeutic advancements, the treatments for breast cancer often come with a plethora of reactions that adversely impact the quality of life for patients.

Nanotechnology has recently emerged as a promising frontier in medical science, offering innovative solutions for bio-imaging, nucleic acid delivery, biosensors, and drug delivery. Nanoparticle-based drug delivery systems have garnered attention for their ability to improve pharmacokinetics, precisely target tumor cells, and mitigate drug resistance. Hybrid nanoparticle development has further enhanced the stability and performance of drug delivery systems by amalgamating the distinct features of various nanoparticles [5]. The potential of nanoparticle-based therapy to address multidrug resistance (MDR) in various cancer types, including breast cancer, has become a focal point of ongoing research [6]. The intersection of nanotechnology and medicine has ushered in a new era for cancer treatment, although further research is essential to fully comprehend the intricate dynamics between these two fields. This study seeks to consolidate fundamental concepts, address existing challenges, and outlining prospective research directions related to the utilization of nanocarrier systems in cancer therapy [7].

Chitosan, a derivative of chitin, has gained prominence as a potential material for drug carrier nanoparticles due to its biodegradability, biocompatibility, and low toxicity. The cationic composition of chitosan facilitates enhanced adherence to mucosal surfaces via electrostatic interactions, leading to improved drug absorption into targeted cells. Chitosan nanoparticles, utilizing encapsulation or direct attachment, have proven to be a robust, safe, and non-toxic alternative for drug delivery. Their ability to encapsulate various compounds has opened up avenues for applications in cancer therapy and other industries such as food, agriculture, cosmetics, and pharmaceuticals [8].

*Alstonia venenata* R.Br. a plant belonging to the Apocynaceae family, has gained prominence in various Indian systems of medicine. With 43 species in the genus Alstonia, *A. scholaris*, *A. boonei*, *A. congensis*, and *A. macrophylla* have demonstrated utility in treating various diseases, owing to their abundance in alkaloids, steroids, triterpenoids, and phenolic compounds [9]. A particular focus is placed on *A. venenata*, a medium-sized shrub endemic to Eastern and Southern India, known for its anti-venom properties. The plant has shown antibacterial efficacy, cytotoxicity against Dalton’s lymphoma ascites (DLA) cells, antioxidant properties, and significant anticancer activity against Ehrlich Ascites carcinoma (EAC) cells, depending on the specific extracts utilized [10,11,12].

Plant-derived secondary metabolites have gained recognition for their potential in cancer treatment, offering natural, eco-friendly alternatives with fewer side effects. These compounds play a crucial role in the development of new anticancer drugs. Natural products, including essential oils and plant extracts, have been explored for their anticancer potential, creating nanoformulations that show preclinical promise. However, translating these findings into clinical applications is hindered by bioavailability challenges [13,14].

Despite the extensive research on natural products and their potential in cancer treatment, there is a notable gap concerning the breast cancer activity of *A. venenata* plant extract. This study seeks to fill that void by evaluating the anticancer potential of *A. venenata* extract loaded with chitosan nanoparticles. This novel approach aims to integrate the strengths of plant-derived compounds and nanotechnology, providing a potential avenue for more effective and targeted breast cancer treatments. The complex interplay between nanocarriers and natural compounds, including the specific impact of chitosan nanoparticles on the efficacy of *A. venenata* extracts, requires further exploration for a comprehensive understanding of their synergistic potential in cancer therapy.

## 2. Materials and Methods

### 2.1. Chemicals

Tripolyphosphate (Cat. No. 7758-29-4, Mumbai, India), glacial acetic acid (Cat. No. 64-19-7, Mumbai, India), Chitosan (Cat. No. 9012-76-4 Mumbai, India), 7,12-dimethylbenz(a)anthracene (Cat No. 68141-56-0, Mumbai, India), ethylenediaminetetraacetic acid (Cat. No. 60-00-4, Mumbai, India), trichloroacetic acid (Cat. No. 76-03-9, Mumbai, India), disodium hydrogen phosphate (Cat. No. 7558-79-4, Mumbai, India), malondialdehyde (Cat. No. 100683-54-3, Mumbai, India), thiobarbituric acid (Cat. No. 504-17-6), magnesium chloride (Cat. No. 7791-18-6, Mumbai, India), sodium fluoride (Cat. No. 7681-49-4, Mumbai, India), hydrazine sulfate (Cat. No. 88491-70-7, Mumbai, India), sodium succinate (Cat. No. 6106-21-4), and potassium ferricyanide (Cat. No. 13746-66-2, Mumbai, India) were purchased from Sigma-Aldrich, Bangalore, India and stored at 4 °C.

### 2.2. Plant Material

The fresh roots of *A. venenata* were collected from Coonoor (GPS, Coordinates: 11°20′42″ N 76°47′42″ E), Tamil Nadu, India. The plant material was authenticated by the Botanical Survey of India, Coimbatore (voucher specimen No: BSI/SRC/5/23/2019/Tech, 3442). The plant samples were thoroughly washed with water, cut into small pieces, and shade-dried for two weeks. The dried samples were powdered using a Wiley mill and stored at 4 °C. The powdered samples were cold-extracted using methanol in the ratio of 1:10 (*w*/*v*) with an orbital shaker at 180 rpm for 24 h at room temperature. The filtrate was concentrated through evaporation at room temperature (20% of the extract yield) and dissolved in methanol to obtain a working solution of 10 mg/10 mL (*w*/*v*).

### 2.3. Preparation and Characterization of Root Methanolic Extract-Encapsulated Chitosan Nanoparticles (RME-ENCNPs)

In silico analysis and in vitro anticancer activity (MTT assay) confirmed the anticancer potential of the root methanolic extract (RME). Consequently, this extract was chosen for encapsulation with chitosan nanoparticles (CNPs) and subjected to characterization, as well as anticancer activity evaluation using an in vivo model.

#### 2.3.1. Preparation of Chitosan Nanoparticles (CNPs)

TPP cross-linked nanochitosan was synthesized using the ionic gelation method [15]. Chitosan (low molecular weight, 0.5% *w*/*v*) was dissolved in 0.5% glacial acetic acid, agitated for 30 min, and left overnight at room temperature. The pH of the solution was set to 5.5 with 0.5 M NaOH. Subsequently, 0.5 mL of tripolyphosphate (0.5% TPP) was added dropwise to the above solution until the color changed to milky white, with stirring at 100 rpm. The nanoparticle suspension was then centrifuged at 10,000 rpm for 10 min at 4 °C temperature. The resulting pellet was collected, lyophilized, and stored at room temperature following the method outlined [16].

#### 2.3.2. Preparation of RME-EnCNPS

Chitosan nanoparticles at five different concentrations (100, 200, 300, 400, and 500 µg/mL) were directly dissolved in root methanolic extract (RME-EnCNPs), followed by the gradual addition of TPP dropwise. The mixture was stirred on a magnetic stirrer for 40 min. Subsequently, it was centrifuged at 15,000 rpm for 10 min, and the resulting pellets were collected. The pellets were washed once or twice with deionized water and then dried at room temperature, following the methodology outlined [17].

#### 2.3.3. Entrapment Efficiency

The encapsulation or entrapment efficiency of the nanoparticles was assessed by measuring the concentration of free RME. In a concise procedure, 2 mL of the RME- EnCNPs dispersion was subjected to ultracentrifugation at 15,000 rpm for 30 min. The supernatant obtained was collected and analyzed for RME-EnCNPs using a UV–visible spectrophotometer at a wavelength of 279 nm, as per the method described by Bagyalakshmi and Haritha [18]. All measurements were conducted in triplicate. The percentage of RME entrapped in the CNPs was calculated using the absorbance values, employing the following equation:

Mass of initially added drug − Mass of free drug EE (%) = (Mass of initially added drug × 100)

### 2.4. In Vitro Drug Release Study

In vitro release profiles of the optimized RME-EnCNPs were generated using the dialysis tube method with a molecular weight cutoff of 12,000–14,000. A known quantity of nanoparticles was dispersed in 2 mL of phosphate-buffered saline (pH 6.8). In addition, the membrane was washed with warm double-distilled water (70 °C) for 1 h and then rinsed thrice with water to eliminate glycerin. Approximately 5 mL of a suspension containing phosphate buffer (pH 6.8) was placed in the dialysis bag, sealed at both ends, and submerged in a beaker containing 100 mL of phosphate buffer (pH 6.8). The sample was stirred at 100 rpm using a magnetic stirrer at room temperature. At specific time intervals (0.5, 1, 1.5, 2, 2.5, and 3 h), a 2 mL aliquot was taken, and absorbance was measured at 450 nm. The spent aliquot was replaced in the dialysis membrane by an equal volume of fresh buffer, as per the method outlined [19].

The concentration of the drug present in the test sample was determined using the following equation, as described in [20]:

Concentration of drug released Drug release [%] = (Total concentration of drug × 100)

To theoretically understand the mechanism of RME release from CNPs, the in vitro drug release data were fitted into different mathematical models, including the zero order, first order, Higuchi model, and Korsmeyer–Peppas model [21].

#### 2.4.1. Drug Release Kinetics

The in vitro dissolution test data were fitted into mathematical models representing (i) fero order, (ii) first order, (iii) Higuchi’s, and (iv) Korsmeyer–Peppas model.

Equations were used to determine the release mechanism and the order of drug release.

#### 2.4.2. Zero Order Kinetics

The zero order kinetics represent the drug release rate, which is independent from its concentration. The equation translating the zero order kinetics is as follows:

Q_t_ = Q_0_ + k_0t_

where Q_t_ is the quantity of RME or drug dissolved in time t, Q_0_ is the initial concentration of the drug in the buffer solution, and K_0t_ is the zero order release constant, which was obtained from the slope of the linear plot of percentage drug release versus time.

#### 2.4.3. First Order Kinetics

The first order kinetics represent the drug release from a system that is concentration-dependent and is expressed as follows:

log C_t_ = log C_0_ − k_1t_/2.303

where C_t_ is the concentration of drug released in time t, C_0_ is the initial concentration of drug present in the nanoparticles, k_1t_ is the first order constant and it was calculated from the slope of the linear plot of the log values of the cumulative percentage of the drug remaining versus time.

#### 2.4.4. Higuchi Model

The Higuchi model describes drug release from an insoluble matrix as the square root of a time-dependent process based on diffusion. The Higuchi model can be expressed as follows:

Q_t_ = k_H_ × t0.5

where Q_t_ is the amount of drug released in time t, and k_H_ is constant, which was obtained from the slope of the linear plot of percentage drug (RME) release versus square root of time.

#### 2.4.5. Korsmeyer–Peppas Model

The Korsmeyer–Peppas model explains drug release from a polymeric system, and can be expressed as follows:

Log_Qt_ = log _k_ + n × log _t_

where k is the kinetic constant and n is the diffusion exponent, a measure of the primary mechanism of drug release. Values of n and k were calculated from the slope and intercept of the linear plot of the log cumulative percentage drug release versus log time.

#### 2.4.6. Correlation Coefficients Analysis (R^2^)

R^2^ analysis helps to determine the regression between selected items. Intercept and slope give the Y2-Y1/X2-X1 value. The R^2^ value of the kinetic models clearly defines the influence of each factor % of drug release, log (cumulative frequency) CF%, and cumulative %.

### 2.5. Characterization of CNPs and RME-EnCNPs

Chitosan nanoparticles (CNPs) and root methanolic extract-encapsulated chitosan nanoparticles (RME-EnCNPs) were subjected to various spectroscopy characterization studies.

#### 2.5.1. Ultraviolet–Visible Spectroscopy (UV–Vis)

To verify the formation of nanoparticles, the solution was scanned in the range of 200–800 nm in a spectrophotometer using a quartz cuvette with water as the reference.

#### 2.5.2. Fourier Transform Infrared (FTIR) Spectroscopy

Fourier transform infrared spectroscopy (FTIR) is a technique used to obtain the infrared spectrum of absorption, emission, and photoconductivity in solids, liquids, and gasses. It is employed to detect various functional groups in a sample, and the spectrum corresponds to specific rotations around carbon atoms. FTIR analysis is performed to analyze the formation of CNPs and RME-EnCNPs. After preparing samples with KBr in pellet form, the spectra were recorded using FTIR at room temperature. The scan range was 4000 to 400 cm^−1^ at a resolution of 4 cm^−1^ [22].

#### 2.5.3. X-Ray Diffraction

XRD analysis was conducted using a Cu Kα X-ray source (λ = 1.5406 Å). This technique is employed to investigate the crystalline nature of chitosan nanoparticles/encapsulated chitosan nanoparticles and to determine nanoparticle size. The analysis was performed within the scanning 2θ range of 20–80° with a scanning speed of 50 S^−1^.

#### 2.5.4. Scanning Electron Microscopy (SEM)

The topography of RME-EnCNPs was examined using a Quanta 400 ESEM/EDAX (FEI). Small amounts of vacuum-dried nanochitosan samples were placed on an SEM stub using double-sided adhesive tape and subjected to sputter coating at 50 mA for 6 min. Subsequently, the stub containing the sample was positioned in the scanning electron microscopy (SEM) chamber, and a photomicrograph was captured at an acceleration voltage of 20 kV.

#### 2.5.5. Zeta Potential

The zeta potential of RME and RME-EnCNPs was analyzed using a laser light scattering-based particle analyzer. The analysis was conducted in triplicate, and average values along with standard deviations were recorded.

### 2.6. Animals and Experimental Design

For the in vivo experiment, 21-day-old female Sprague Dawley rats weighing 120–140 g were utilized, and they were procured from the National Institute of Nutrition, Hyderabad, India. All the rats were housed at a room temperature of 22 ± 3 °C with a 12 h light/12 h dark cycle in the animal house. Throughout the study, the animals were provided with a commercial pellet diet and had free access to water, adhering to the guidelines of the Committee for the Purpose of Control and Supervision of Experiments on Animals (CPCSEA), New Delhi.

The experiment was conducted following the ethical guidelines and after obtaining clearance from the Institutional Animal Ethics Committee (IAEC) under IAEC No. KMCRET/ReRc/Ph.D/20/2021. The seven animal groups were separated, with each group being assigned eight animals. The animal treatment groups were as follows:

Group I Control rats given only saline (p.o.)
Group II Rats given DMBA only 25 mg/kg (i.p)
Group III Rats treated with DMBA + tamoxifen 10 mg/kg (standard control, p.o.)
Group IV Rats treated with DMBA + RME 250 mg/kg (p.o.)
Group V Rats treated with DMBA + RME 500 mg/kg (p.o.)
Group VI Rats treated with DMBA + RME-EnCNPs 250 mg/kg (p.o.)
Group VII Rats treated with DMBA + RME-EnCNPs 500 mg/kg (p.o.).

After completing the induction and treatment period, the animals were sacrificed and their tissues were collected for the evaluation of the estimation of the tumor parameters, enzymatic antioxidant and non-enzymatic antioxidant activities, glycolytic and non-glycolytic enzymes, estimation of mitochondrial TCA cycle enzymes, and histopathology analysis. The blood samples were used for biochemical analysis.

#### 2.6.1. Acute Toxicity Study

To assess the safety of the plant extracts (RME and RME-EnCNPs), an acute oral toxicity analysis was conducted following the guidelines of the Organization for Economic Co-operation and Development (OECD) for testing chemicals [23]. The analysis received approval from the Institutional Animal Ethics Committee (IAEC). For this experiment, healthy female Sprague Dawley rats weighing between 120 and 140 g were selected and divided into 6 groups, with 3 animals in each group. The rats were housed in plastic cages under a 12 h light and dark period, with room temperature maintained at 22 ± 3 °C and humidity between 40 and 60%, ensuring proper ventilation. Oral doses of 500, 1000, and 2000 mg/kg of RME and RME-EnCNPs were administered to each group. Throughout a 14-day observation period, the animals were monitored for changes in behavior, function, and morphology. Observations included an assessment of toxicological symptoms such as alertness, grooming, touch response, torch response, pain response, tremors, convulsion, righting reflex, gripping strength, pinna reflex, corneal reflex, writhing, pupils, urination, salivation, and skin color [24].

#### 2.6.2. Breast Cancer Induction by Chemical Carcinogen

In this study, 7,12-dimethylbenz(a)anthracene (DMBA) was employed to induce a chemical model of breast cancer in female rats. This model was utilized to investigate the chemopreventive effects of RME and RME-EnCNPs, exploring their potential in treating and preventing breast cancer. DMBA, an aromatic pollutant, is recognized as a carcinogen capable of inducing cancer in humans and is commonly used to induce mammary gland tumors in rats [25].

Female Sprague Dawley rats, 30 days old and weighing approximately 120 to 140 g, were used for the induction of breast cancer by intra-peritoneal injection of DMBA (25 mg/mL dissolved in olive oil) over a 16-week period. After the development of breast tumors, the rats were treated with RME and RME-EnCNPs at 2 concentrations, 250 and 500 mg/kg body weight, administered orally for a duration of 4 weeks.

#### 2.6.3. Estimation of Body Weight

Body weights were estimated for all the groups of animals at the end of 20 weeks using electrical balance [26].

#### 2.6.4. Animal Sacrifice

At the end of the 20th week of treatment, all the rats were anesthetized with diethyl ether and euthanized by decapitation. The animals were subjected to overnight fasting before the sacrificial procedure. Blood was collected and transferred into tubes containing ethylenediaminetetraacetic acid (EDTA) for hematological parameters, while serum was separated by centrifugation at 3000 rpm for 15 min and stored at −20 °C for biochemical assays. Breast tumors were removed, counted, weighed, and measured using a 1 mm precision caliper. Additionally, the mammary gland, liver, kidney, and uterus were collected and immediately fixed in a 10% formalin solution.

#### 2.6.5. Estimation of Tumor Parameters

The tumor volume was measured using the following formula: Tumor volume (cm^3^) = Length × Weight × Height × π/6. The tumor weight was estimated for all the animal groups [27] using the formula: Tumor weight (g) = Length (cm) × Width (Cm^2^)/2. The tumor burden was measured by the number of tumors formed in each animal [27], calculated using the following formula: Tumor burden (g) = Sum of the total relative tumor mass in a group. The tumor incidence percentage, calculated based on the presence of tumors, was determined by the following formula [27]: Tumor Incidence (%) = (Rat with tumors/Total number of rats) × 100. These parameters were utilized for the assessment of the effects of the treatments on tumor growth and incidence in accordance with the methodologies described [27]. 

#### 2.6.6. Biochemical Assessment of Blood 

A biochemical analysis of blood measures the amount of chemical substances released by body tissues during the metabolism of specific substances. This analysis provides valuable information about the function of organs such as the liver, kidneys, and others [27].

The separated serum from all seven groups of animals was used for the estimation of various parameters, including protein, bilirubin, serum glutamic oxaloacetic transaminase (SGOT), Serum Glutamic Pyruvic Transaminase (SGPT), alkaline phosphatase (ALP), estrogen hormone, progesterone hormone, total cholesterol, triglycerides, HDL-cholesterol, urea, uric acid, and creatinine. These analyses were performed using a semi-auto analyzer (RIELE Photometer 5010 V5+, RIELE, New Delhi, India), and standard enzymatic kits were procured from Piramal Healthcare Limited, Lab Diagnostic Division, Mumbai, India.

#### 2.6.7. Enzymatic Antioxidant and Non-Enzymatic Antioxidant Activities

##### Estimation of Protein

The estimation of protein was conducted by using 0.1 mL of the homogenate mixed with 0.9 mL of water. Subsequently, 4.5 mL of the alkaline copper sulfate reagent was added, and the mixture was allowed to stand at room temperature for 10 min. To this solution, 0.5 mL of Folin’s reagent was added. After 20 min, the color produced was measured at 640 nm. The concentration of protein present was expressed as either mg/g tissue or mg/dL, following the methodology outlined [28].

##### Estimation of Superoxide Dismutase (SOD)

In this assay, 0.5 mL of supernatant tissue homogenate was added to a test tube. To this, 1.5 mL of carbonate buffer (pH 7.5), 0.5 mL of 0.1 mM EDTA, and 0.4 mL of epinephrine were added, and the absorbance reading was taken at 480 nm, following the procedure outlined [29]. The activity of SOD was expressed as units/min/mg protein, where one unit of the enzyme is defined as the amount that inhibits the rate of adrenaline autoxidation by 50%.

##### Estimation of Catalase

To analyze catalase activity, 1 mL portions of the reaction mixture were withdrawn and transferred to 2 mL of dichromate/acetic reagent at 1 min intervals. The mixture was then incubated for 30 min, and the optical density (OD) was measured at 570 nm. The activity of catalase was expressed as µmoles of H_2_O_2_ consumed per mg protein, following the method described [30].

##### Estimation of Glutathione Peroxidase (GPx)

To estimate GPx activity, a reaction mixture consisting of 0.2 mL each of EDTA, sodium azide and H_2_O_2_, 0.4 mL of phosphate buffer, and 0.1 mL homogenate/mitochondria was incubated at 37 °C at different time intervals. The reaction was halted by adding 0.5 mL of trichloroacetic acid (TCA), and the tubes were then centrifuged at 2000 rpm. To 0.5 mL of the supernatant, 4 mL of disodium hydrogen phosphate and 0.5 mL of DTNB were added, and the developed color was immediately measured at 420 nm. The activity of GPx was expressed as µmoles of glutathione oxidized per mg protein, following the method described [31].

##### Estimation of Reduced Glutathione (GSH)

The estimation of reduced glutathione (GSH) activity was carried out using 1 mL of the tissue homogenate, which was then treated with 1 mL of trichloroacetic acid (TCA) to precipitate proteins. The precipitate was removed by centrifugation. To 0.5 mL of the supernatant, 2 mL of DTNB was added, and the total volume was adjusted to 3 mL with phosphate buffer. The absorbance was read at 412 nm. The level of glutathione was expressed as µg/mg protein, following the methods outlined [32].

##### Lipid Peroxidation

In this method, malondialdehyde (MDA) and other thiobarbituric acid reactive substances (TBARS) were estimated by their reactivity with thiobarbituric acid (TBA) under acidic conditions to generate a pink-colored chromophore, which was then measured at 535 nm (pH 7.5). For the assay, 0.1 mL of tissue homogenate in Tris-HCl buffer (pH 7.5) was treated with 2 mL of TBA-HCl-TCA reagent in a 1:1:1 ratio (thiobarbituric acid 0.37%, 0.25 N HCl, and 15% TCA). The mixture was placed in a water bath for 15 min, then cooled. The absorbance of the clear supernatant was measured against a reference blank at 535 nm. The level of lipid peroxides was expressed in moles of MDA formed per mg protein [33].

#### 2.6.8. Glycolytic and Non-Glycolytic Enzymes

The pooled samples from the seven groups of animals were subjected to assays for glycolytic enzymes (hexokinase and aldolase) and non-glycolytic enzymes (glucose 6-phosphate and fructose-1,6-diphosphatase) as described in [34].

##### Hexokinase

Hexokinase activity was measured based on the amount of glucose utilized after the addition of ATP. The incubation mixture consisted of 2.5 mL Tris-HCl buffer (pH 8.0), 1 mL of glucose, 0.5 mL ATP, 0.1 mL each of MgCl_2_ and sodium fluoride, and 0.5 mL each of KH_2_PO_4_ and KCl. This mixture was pre-incubated at 37 °C for 5 min at room temperature. The reaction was initiated by adding 2 mL of glycolytic enzyme. At zero time, 1 mL aliquot of the reaction mixture was removed and added to tubes containing 1 mL of 10% trichloroacetic acid (TCA). After a 30 min incubation, another 1.0 mL aliquot of the reaction mixture was added to a separate set of tubes, and the reaction was stopped by the addition of 1.0 mL of TCA. After the samples were precipitated and centrifuged, the supernatants were used for the estimation of glucose by the ortho-toluidine method at 340 nm. The level of hexokinase was expressed as nmoles of glucose 6-phosphate per min per mg protein, following the method described [35].

##### Aldolase

For the aldolase assay, the incubation sample contained 0.25 mL of fructose-1,6-diphosphate, 0.25 mL of hydrazine sulfate, 1 mL of Tris-HCl buffer (pH 8.6), and the required amount of preparation to make it to 2 mL. This mixture was incubated at 37 °C for 15 min. The reaction was terminated by the addition of 10% trichloroacetic acid (TCA), and the tubes were centrifuged. An aliquot of the supernatant was transferred to tubes containing 1 mL of 0.75 N NaOH. The tubes were left at room temperature for 10 min. To this, 1 mL of dinitrophenyl hydrazine reagent was added, and the mixture was incubated at 37 °C for 60 min. The color developed after the addition of 7 mL of 0.75 N NaOH and the solution was read using a green filter at 540 nm. A color development curve was prepared with aliquots of a standard DL-glyceraldehyde solution by treating them in a similar manner. The enzyme activity was expressed as nmoles of glyceraldehyde formed per min per mg protein, following the method described [36].

##### Fructose-1,6-Diphosphatase

The assay medium, in a final volume, contained 1.2 mL of Tris-HCl buffer (pH 7.0), 0.1 mL of fructose-1,6-diphosphatase solution, 0.25 mL of MgCl2, 0.1 mL of KCl solution, 0.25 mL of EDTA solution, and 0.1 mL of enzyme. The incubation was carried out at 35 °C for 15 min, and the reaction was terminated by the addition of 1 mL of trichloroacetic acid (TCA). After centrifugation, the phosphorus content of the supernatant was estimated. The enzyme activity is expressed as nmoles of inorganic phosphate (Pi) liberated per minute per mg protein, following the method described [36].

##### Glucose-6-Phosphate

Glucose-6-phosphate was assayed according to the method described by King (1965b) [37]. The incubation mixture comprised 0.3 mL of citrate buffer (pH 6.5), 0.5 mL of glucose-6-phosphate, and 0.2 mL of enzyme solution. Incubation was carried out at 37 °C for 60 min. The reaction was terminated by the addition of 1 mL of 10% trichloroacetic acid (TCA) solution. After centrifugation, the phosphorus content in the supernatant was estimated by the method of Fiske and Subbarow [36]. The enzyme activity is expressed as nmoles of inorganic phosphate (Pi) liberated per minute per mg protein.

#### 2.6.9. Assessment of Mitochondrial TCA Cycle Enzyme Activity

##### Isocitrate Dehydrogenase

The activity of isocitrate dehydrogenase was assayed according to the method outlined by King [37]. To 0.1 mL of Tris-HCl (pH 7.5), 0.2 mL of trisodiumisocitrate, 0.3 mL of manganese chloride, 0.2 mL of mitochondrial suspension, and 0.2 mL of NADP+ (0.2 mL of water for control) were added. After 60 min of incubation, 1 mL of 2,4-Dinitrophenylhydrazine was added followed by 0.5 mL of EDTA, and the mixture was kept at room temperature for 20 min. Then, 10 mL of NaOH was added, and the color developed was read at 390 nm. A standard containing α-ketoglutarate was run simultaneously. The isocitrate dehydrogenase activity was expressed as nmoles of α-ketoglutarate liberated per min per mg protein.

##### Succinate Dehydroganase

The activity of succinate dehydrogenase (SDH) in the heart mitochondrial fraction was assayed using the method outlined [38]. The reaction mixture contained 1.0 mL of phosphate buffer, 0.1 mL of EDTA, 0.1 mL of sodium cyanide, 0.1 mL of bovine serum albumin, 0.3 mL of sodium succinate, 0.2 mL of potassium ferricyanide, and was made up to 2.8 mL with distilled water. The reaction was initiated by the addition of 0.2 mL of mitochondrial fraction. The change in optical density was recorded at 15 s intervals for 5 min at 420 nm. The succinate dehydrogenase activity was expressed as nmoles of succinate oxidized per minute per mg protein.

##### Malate Dehydrogenase

The activity of malate dehydrogenase (MDH) in the heart mitochondrial fraction was assayed using the method described [39]. The reaction mixture consisted of 0.75 mL of phosphate buffer, 0.15 mL of reduced nicotinamide adenine dinucleotide (NADH), and 0.75 mL of oxaloacetate. The reaction was carried out at 25 °C and was initiated by the addition of 0.2 mL of mitochondrial fraction. The control tubes contained all reagents except NADH. The change in optical density at 340 nm was measured for 2 min at intervals of 15 s using a Systronics UV–visible spectrophotometer (Systronics, Ahmedabad, India). The enzyme activity was expressed as nmoles of NADH oxidized per minute per mg of total protein under the given incubation conditions.

##### Quantification of Hematological Parameters

Different hematological parameters were assessed using a Mindray BC-2800 Auto Hematology Analyzer from Shenzhen Mindray Bio-Medical Electronics Co., Ltd. (Shenzhen, China). After the treatment period, the animals were anesthetized with ketamine hydrochloride, and blood was collected from the retro-orbital sinus using a capillary tube into a centrifugation tube containing EDTA for hematological parameters. It was allowed to clot at room temperature, and serum was separated by centrifugation at 10,000 rpm for 10 min and utilized for hematological parameters such as red blood cell (RBC) count, white blood cell (WBC) count, hemoglobin, packed cell volume (PCV), polymorphs, lymphocytes, eosinophils, and mean corpuscular hemoglobin (MCH) [40].

#### 2.6.10. Histopathology Analysis

Small pieces of rat breast tissues were fixed in 10% formalin for 24 h. Subsequently, the tissues underwent dehydration through ethanol, and then they were embedded in paraffin. Sections of 5 µm thickness were cut and stained with hematoxylin and eosin for histopathological investigation under a light microscope [41].

#### 2.6.11. Statistical Analysis

The results were expressed as the mean ± SEM. The results obtained from the present study were analyzed by using one-way ANOVA followed by Dunnett’s multiple comparison tests. Data were computed for statistical analysis by using GraphPad Prism version 5.00 for Windows, Graph Pad Software version 8, San Diego, CA, USA, www.graphpad.com (accessed on 15 March 2022) and compared with the vehicle control group. The *p* values (*p* < 0.05) were considered statistically significant.

## 3. Results

### 3.1. The Entrapment Efficiency (EE) of RME-EnCNPs

**Figure 1:**
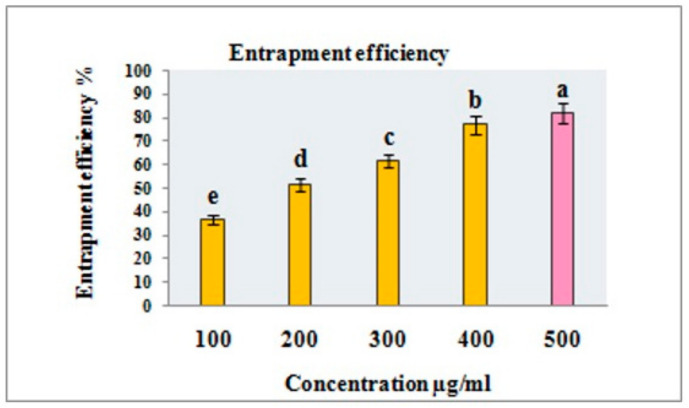
Entrapment efficiency of root methanolic extract-encapsulated chitosan nanoparticles (RME-EnCNPs). Effects a, b, c, d, and e were significant with consideration of probability *p* < 0.05. Values are means ± standard deviation of three replicates.

The entrapment efficiency (EE) of RME-EnCNPs was assessed with three replicates. The impact of different concentrations of RME on EE is presented in Figure 1. Chitosan nanoparticles exhibited an escalating trend in encapsulation effectiveness with the rise in RME concentrations. Among the various RME concentrations tested (100, 200, 300, 400, and 500 µg/mL), the highest entrapment efficiency (81.81%) was observed at 500 µg/mL (Figure 1).

**Figure 2:**
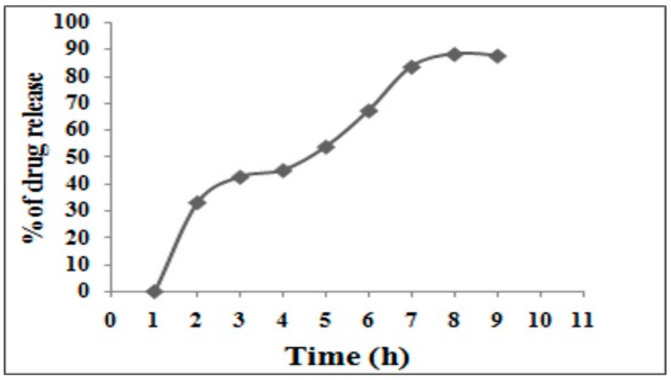
Drug release percentage of RME-EnCNPs.

The in vitro drug release profile of the RME-EnCNPs demonstrated a slow and sustained release, with 50% of the drug being released within 2 h. Subsequently, after 5 h, 87.8% of the drug was released, as illustrated in Figure 2. The initial release observed within the first hours could be attributed to the release of drug molecules from the surface of chitosan nanoparticles. To comprehend the drug release mechanism, RME-EnCNPs underwent analysis using various mathematical models, including zero order, first order, Higuchi, and Korsmeyer–Peppas models.

**Figure 3:**
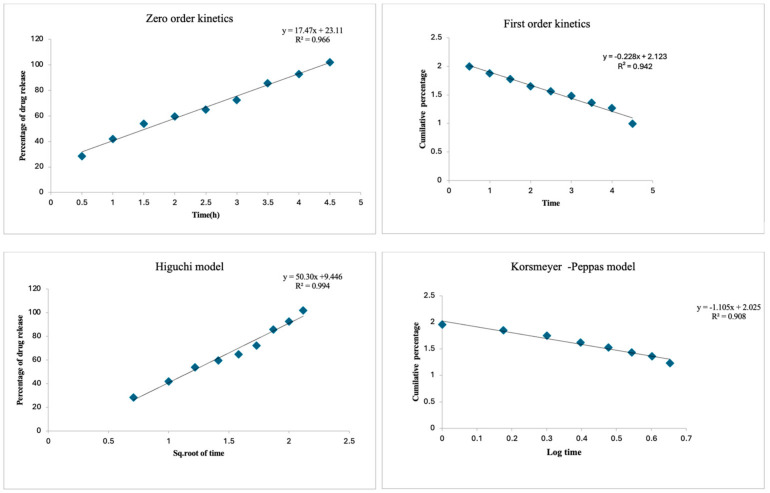
In vitro drug release kinetic models.

The correlation coefficient values for these kinetic models are presented in Table 1; they help determine the most appropriate model for the slow and sustained drug release. In this study, the kinetic models of zero order, first order, Higuchi, and Korsmeyer–Peppas displayed good linearity, with R^2^ values of 0.966, 0.942, 0.994, and 0.908, respectively. Notably, the Higuchi model exhibited the highest R^2^ value of 0.994, confirming its suitability for describing the drug’s delivery nature, as depicted in Figure 3.

### 3.2. Characterization of RME-EnCNPs

#### 3.2.1. Ultraviolet–Visible Spectroscopy (UV–Vis) Characterization

**Figure 4:**
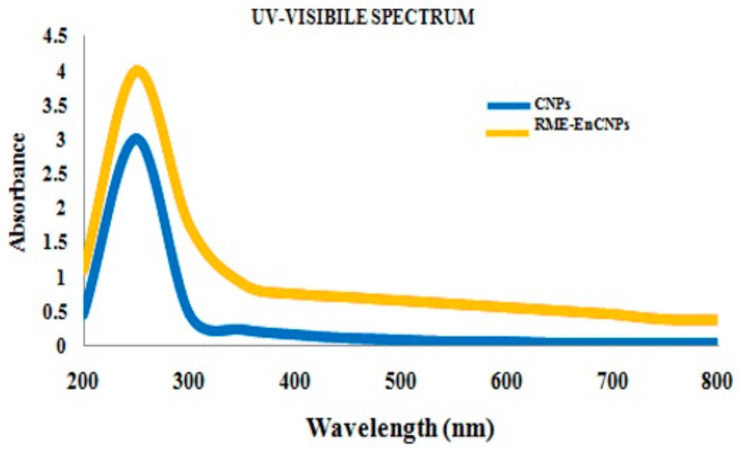
UV absorption spectra of chitosan nanoparticles (CNPs) and root methanolic extract-encapsulated chitosan nanoparticles (RME-EnCNPs).

A concentration of 500 µg/mL of the RME-EnCNPs was chosen for comprehensive characterization studies. The encapsulation process of the RME-EnCNPs (500 µg/mL) and CSNPs was monitored and analyzed using a UV–vis spectrophotometer within the range of 200–800 nm (Figure 4). Both samples exhibited a single, broad, and sharp peak with a maximum absorbance wavelength at 250 nm. The consistent absorption maxima indicated that RME was effectively loaded within the chitosan nanoparticles (CNPs).

#### 3.2.2. Fourier Transform Infrared Spectroscopy (FTIR) Characterization

**Figure 5:**
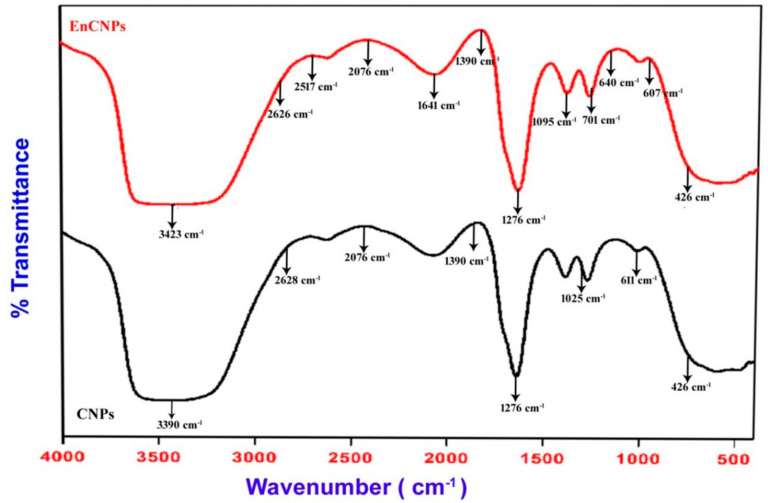
FTIR Spectra of chitosan nanoparticles (CNPs) and root methanolic extract-encapsulated chitosan nanoparticles (RME-EnCNPs).

The FTIR spectrum (Figure 5) of chitosan displays characteristic bands attributed to the NH2 group’s stretching vibration at the wavelength of 3390.24 cm^−1^, C–O stretching at the wavelength of 1025.94 cm^−1^, and the C-H vibration bond at the wavelength of 611.324 cm^−1^. In the spectrum of the RME-EnCNPs (Figure 5), three distinctive bands unique to RME were observed: O-H in alcohols and phenols at 3423.03 cm^−1^, C-W waging in alkyl halides at 1095.37 cm^−1^, and stretching vibration of CH bend out of plane at 640.251 cm^−1^.

#### 3.2.3. XRD Analysis

**Figure 6:**
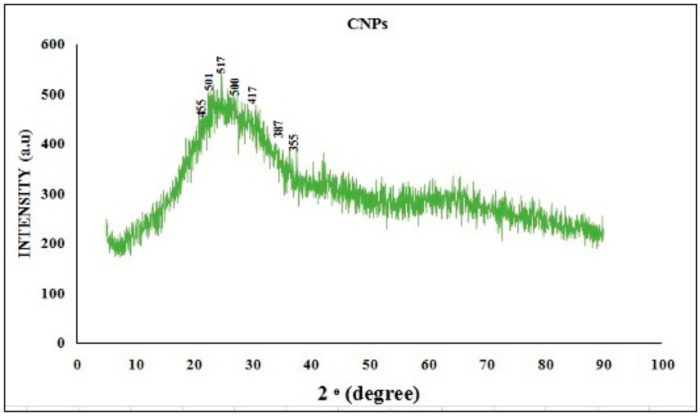
X-ray diffraction pattern of chitosan nanoparticles (CNPs) at 2θ (degree).

**Figure 7:**
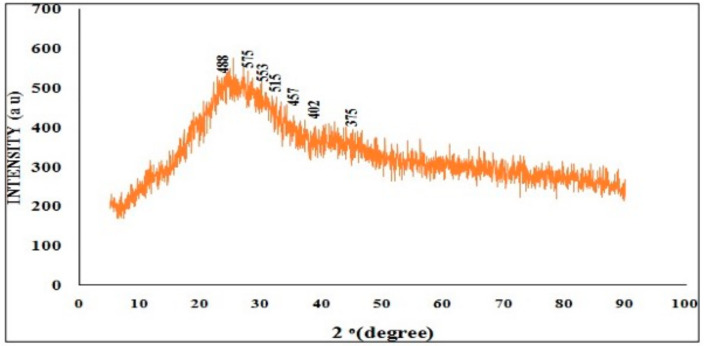
X-ray diffraction pattern of root methanolic extract-encapsulated chitosan nanoparticles (RME-EnCNPs) at 2θ (degree).

The XRD patterns of the CNPs and RME-EnCNPs at 2θ in the range of 10–100° are depicted in Figure 6 and Figure 7. The results indicate that RME-EnCNPs exhibit characteristics of both chitosan and RME. Both samples display an amorphous nature in their XRD patterns. The X-ray diffraction pattern of CNPs revealed prominent peaks in the 2θ range, corresponding to various crystal planes, including 20.49° (455), 22.50° (541), 27.50° (517), 30.50° (500), 35.50° (417), 37.49° (387), and 40.50° (355). In comparison, RME-EnCNPs exhibited crystal peaks at 22.50° (488), 24.49° (575), 28.49° (553), 31.50° (515), 36.48° (457), 39.50° (402), and 44.49° (375). Despite these crystalline peaks, both samples retained an amorphous nature in their XRD patterns. The broad diffraction peak observed in RME-EnCNPs is attributed to the loading of RME onto chitosan nanoparticles. The average size of CNPs and RME-EnCNPs was determined as 79 nm and 83 nm, respectively, utilizing the Debye–Scherrer equation. This size difference aligns with the successful loading of RME onto chitosan nanoparticles, contributing to the observed broad diffraction peak in the XRD pattern of the RME-EnCNPs.

#### 3.2.4. Scanning Electron Microscope (SEM) Analysis

**Figure 8:**
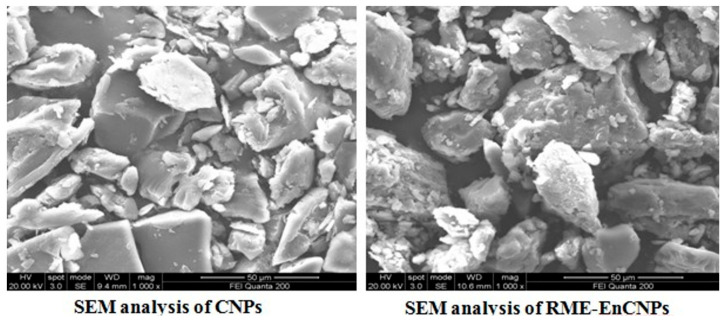
SEM images of chitosan nanoparticles (CNPs) and root methanolic extract-encapsulated chitosan nanoparticles (RME-EnCNPs).

Figure 8 depicts the SEM images of CNPs and RME-EnCNPs at a 10 μm scale bar. The SEM images confirm the successful formation of chitosan nanoparticles. In the SEM image of the CNPs, most particles appeared to be aggregated. However, in the SEM image of the RME-loaded CNPs, a uniform and spherical morphology was observed. The average size of the CNPs and RME-EnCNPs was measured as 79 nm and 83 nm, respectively. It is worth noting that the size of the nanoparticles increased after encapsulating RME onto chitosan nanoparticles.

#### 3.2.5. Zeta Potential Analysis

**Figure 9:**
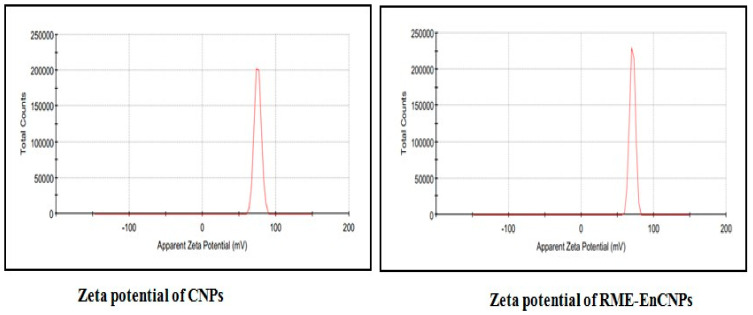
Zeta potential images of chitosan nanoparticles (CNPs) and root methanolic extract-encapsulated chitosan nanoparticles (RME-EnCNPs).

The zeta potential values for CNPs and RME-EnCNPs are illustrated in Figure 9, with values of 33.5 mV and 40.6 mV, respectively.

### 3.3. Evaluation of Acute Oral Toxicity

RME and RME-EnCNPs were utilized to assess acute toxicity. Visual parameters, including alertness, grooming, touch response, torch response, pain response, tremors, convulsions, righting reflex, gripping strength, pinna reflex, corneal reflex, writhing, pupils, urination, salivation, and skin color, were observed at specific intervals over a two-week period. Throughout the observation period, the rats did not exhibit any abnormal signs, behavioral changes, body weight fluctuations, or macroscopic findings. Furthermore, there were no instances of mortality recorded for the administered doses (500, 1000, 2000 mg/kg) at the conclusion of the 14-day observation period.

### 3.4. Assessment of Physical Parameters (Body Weight) of DMBA-Induced Mammary Cancer in Female Sprague Dawley Rats

In general, the body weight of the animals treated with RME and RME-EnCNPs showed an increase compared to DMBA (135 ± 1.0 g)-treated animals. Of the two doses of RME and RME-EnCNPs, the higher dose, i.e., 500 mg/kg, exhibited enhanced body weight (185 ± 1.9 g) (Table 2).

#### 3.4.1. Analysis of Tumor Weight, Volume, Incidence, and Burden

During this period, a notable reduction (*p* < 0.001) in tumor weight (4.42 ± 0.878 mg/kg), volume (1.3 ± 0.024 mg/kg), burden (2.84 ± 0.577 mg/kg), and incidence (2.21 ± 0.333 mg/kg) was observed in Group VII (DMBA + RME-EnCNPs 500 mg/kg) in comparison to Group II (DMBA 25 mg/kg), as indicated in Table 3.

#### 3.4.2. Biochemical Parameters

In the current study, a noteworthy reduction (*p* < 0.001) in total bilirubin (1.3 ± 0.289 mg/dL), SGOT (40 ± 4.04 u/L), SGPT (36.7 ± 0.82 u/L), and ALP (130 ± 4.62 u/L) was observed in Group VII (DMBA + RME-EnCNPs 500 mg/kg) during this period compared to Group II (DMBA 25 mg/kg). Additionally, hormonal analysis revealed an increase in the levels of estrogen and progesterone in DMBA-induced breast cancer rats (Group II).

These two hormones exhibited a decrease from Group III to Group VII, with a more significant reduction (*p* < 0.001) observed in RME-EnCNPs 500 mg/kg (estrogen: 50.1 ± 1.98 pg/mL; progesterone: 43.9 ± 0.39 ng/mL). A similar trend was observed for total cholesterol, where an increased level was noted in Group II (DMBA-induced breast cancer rats: 215 ± 8.6 mg/dL). The total cholesterol levels decreased from Group III to Group VII, with a more significant reduction (*p* < 0.001) observed in RME-EnCNPs 500 mg/kg (151 ± 2.89 mg/dL). The same pattern was noted for total triglycerides in the treated animals, with an increased level observed in Group II (DMBA-induced breast cancer rats: 183 ± 3.7 mg/dL) (Table 4).

The total triglycerides exhibited a decrease from Group III to Group VII, with a more significant reduction (*p* < 0.001) observed in RME-EnCNPs 500 mg/kg (98 ± 2.34 mg/dL). Additionally, a significant increase (*p* < 0.001) in total HDL cholesterol was noted in Group VII (DMBA + RME-EnCNPs 500 mg/kg: 65.2 ± 0.318 mg/dL) during this period compared to Group II (DMBA 25 mg/kg) (Table 4).

In the present study, all three kidney markers (urea, uric acid, and creatinine) showed elevated levels in Group II (DMBA 25 mg/kg: 76 ± 4.07 mg/dL; 44.2 ± 0.34 mg/dL; 28.3 ± 0.52 mg/dL) compared to other groups treated with RME and RME-EnCNPs (200 and 500 mg/kg), as presented in Table 4.

#### 3.4.3. In Vivo Antioxidant Activity

In the present study, DMBA-induced rats exhibited a significant reduction in total protein, SOD, catalase, GPx, and GSH (81.1 ± 0.06 mg/dL; 0.32 ± 0.06 Unit/min/mg protein; 1.01 ± 0.07 Unit/min/mg protein; 5.02 ± 0.46 nmol of glutathione peroxidase/min/mg protein and 0.36 ± 0.04 nmol of glutathione hydroxidase/min/mg protein), except for LPO, which significantly increased (23.41 ± 0.04 nmol of MDA/mg protein). The trend was found to be the inverse from Group III to Group VII. However, a more significant reduction (*p* < 0.001) was observed in Group VII (RME-EnCNPs 500 mg/kg treated animals), as shown in Table 5.

#### 3.4.4. Enzymes Involved in Glycolysis and Non-Glycolytic Pathways

**Figure 10:**
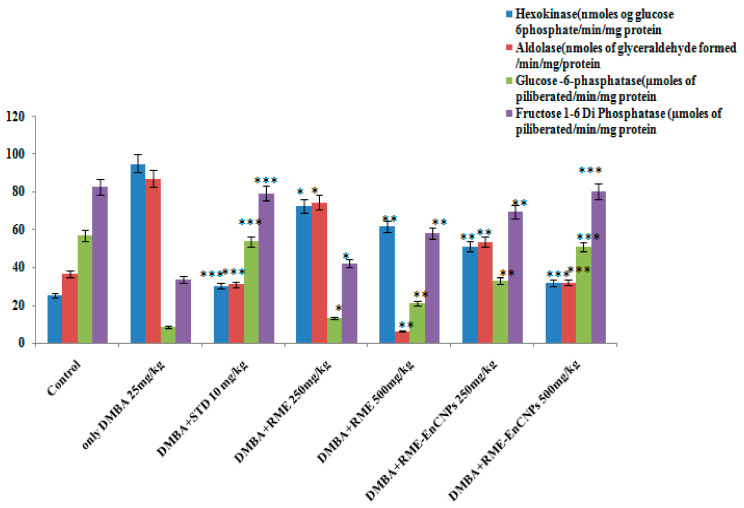
Effect of RME and RME-EnCNPs on hexokinase, aldolase, glucose-6-phosphatase, fructose 1-6-Di phosphatase levels in female Sprague Dawley rats. Results expressed by triplicate means (*n* = 3) ± standard error of mean (SEM). Statistical significance determined with Dunnett’s t-distribution with probability value, *** *p* < 0.001, ** *p* < 0.01, * *p* < 0.05 calculated by comparing treated group with control group. DMBA-7, 12-Dimethylbenz[a]anthracene; STD, standard (tamoxifen); RME, root methanolic extract; RME-EnCNPs, root methanolic extract-encapsulated chitosan nanoparticles.

In the present study, glycolytic enzymes such as hexokinase and aldolase were found to be increased in the DMBA-induced breast cancer rats compared to RME and RME-EnCNPs (200 and 500 mg/kg treated rats). Conversely, the trend was the inverse for non-glycolytic enzymes such as glucose 6-phosphate and fructose 1,6-diphosphatase analyzed in DMBA breast cancer-induced rats and those treated with RME and RME-EnCNPs. However, there was a significant decrease (*p* < 0.001) observed in glucose 6-phosphatase (31.8 ± 0.32 µmoles of glucose 6-phosphatase/min/mg protein) and glyceraldehyde (32.1 ± 0.07 µmoles of glyceraldehyde’s/min/mg protein) in Group VII (RME-EnCNPs 500 mg/kg treated animals). Simultaneously, an increase (53.9 ± 3.46 µmoles of glucose 6-phosphatase/min/mg protein; 51.3 ± 3.52 fructose 1,6-diphosphatase/min/mg protein) was observed for the same group, as depicted in Figure 10.

#### 3.4.5. Estimation of Mitochondrial TCA Cycle Enzymes

**Figure 11:**
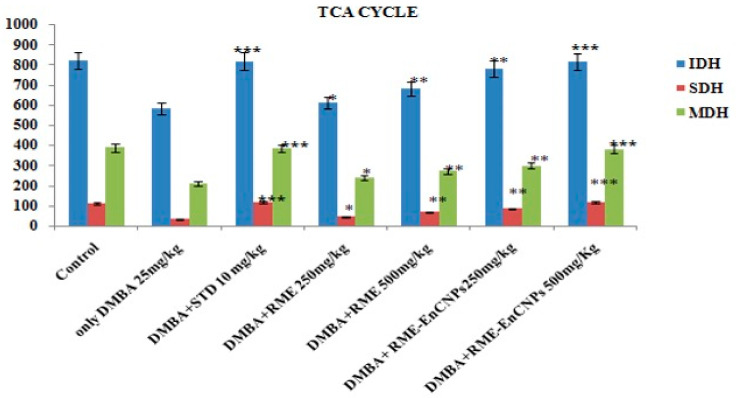
Effect of root methanolic extract (RME) and root methanolic extract-encapsulated chitosan nanoparticles (RME-EnCNPs) on isocitrate dehydrogenase (IDH), succinate dehydrogenase (SDH), and malate dehydrogenase (MDH) levels in female Sprague Dawley rats. Results expressed by triplicate means (*n* = 3) ± standard error of mean (SEM). Statistical significance determined with Dunnett’s t-distribution with probability value, *** *p* < 0.001, ** *p* < 0.01, * *p* < 0.05 calculated by comparing treated group with control group. DMBA-7, 12-Dimethylbenz[a]anthracene; STD, standard (tamoxifen); RME, root methanolic extract; RME-EnCNPs, root methanolic extract-encapsulated chitosan nanoparticles.

In the present study, TCA cycle enzymes such as isocitrate dehydrogenase (IDH), succinate dehydrogenase (SDH), and malate dehydrogenase (MDH) were found to be decreased in the DMBA-induced breast cancer animals (585 ± 15.5 µmoles of α- ketoglutarate/min/mg protein; 35.4 ± 0.303 µmoles of succinate oxidized/min/mg protein; 213 ± 3.52 µmoles of NADH oxidized/min/mg protein). However, RME-EnCNPs 500 mg/kg treated animals showed a significant increase (*p* < 0.001) in IDH (817 ± 6.33 µmoles of α-ketoglutarate/min/mg protein), SDH (115.8 ± 0.641 µmoles of succinate oxidized/min/mg protein), and MDH (213 ± 3.52 µmoles of NADH oxidized/min/mg protein), as illustrated in Figure 11.

#### 3.4.6. Estimation of Hematological Parameters

The analysis of hematological parameters, including RBC, WBC, HB, PCV, polymorphs, lymphocytes, monocytes, eosinophils, and MCH, was conducted in the in rats with DMBA-induced mammary tumors. The results are presented in Table 6. A significant increase (*p* < 0.001) in RBC, HB, PCV, and MCH was observed in Group VII (DMBA + RME-EnCNPs 500 mg/kg: 16.07 ± 0.109; 14.3 ± 0.265; 37.3 ± 0.882; 32.6 ± 0.99) during this period compared to Group II (DMBA 25 mg/kg), which showed a decrease in these parameters (1.12 ± 0.139; 1.9 ± 0.491; 3.8 ± 1.23; 8.4 ± 1.07). Additionally, there was a significant decrease (*p* < 0.001) in lymphocytes, monocytes, and eosinophils in Group VII (DMBA + RME-EnCNPs 500 mg/kg: 88 ± 3.21; 54.3 ± 1.45; 38.7 ± 0.882) compared to Group II (DMBA 25 mg/kg: 52.7 ± 3.28; 6.67 ± 1.45; 8.4 ± 1.07), which exhibited decreased levels of these parameters.

#### 3.4.7. Microscopic Histological Analysis

##### Histology of DMBA-Induced Mammary Tumor of Female Sprague Dawley Rats

Histopathological examinations of DMBA-induced mammary tissue sections from the experimental groups were conducted. The histomorphology of the mammary glands in the normal rat (Group I control) showed no evidence of malignancy, exhibiting normal ductal epithelial cells, nipple areola, and cytoplasm. In Group II (DMBA treatment), malignant epithelial cells with hyperchromasia and nuclear-cytoplasmic ratio, along with pleomorphism, were observed.

Group III (DMBA + standard—tamoxifen treatment) displayed nipple areola with hyperplastic glands, a reduction in tumor fibrosis, and the presence of elastosis with stroma and acinus. Group IV (DMBA + RME 250 mg/kg) exhibited neoplastic glands and fibrosis with myxoid degeneration, accompanied by individual cells of necrosis. Group V (DMBA + RME 500 mg/kg) showed non-neoplastic glands and acinus with fibrosis, and occasional areas of necrosis were seen.

**Figure 12:**
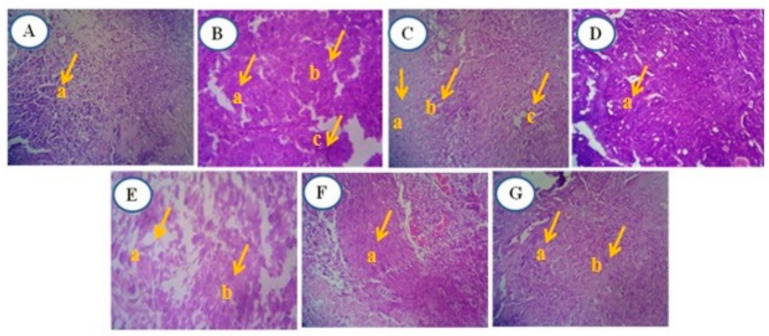
Histology of DMBA-induced mammary tumors of female Sprague Dawley rats. (**A**–**G**) Shows histopathological analysis of mammary tumors of control and experimental groups. Group (**A**): Control showed (a—arrow) normal architecture. Group (**B**): DMBA-induced cancer showed (a—arrow) malignant epithelial cells, (b—arrow) hyperchromasia, (c—arrow) nuclear cytoplasmic ratio and pleomorphism. Group (**C**): DMBA + standard (tamoxifen) treated animals showed (a—arrow) nipple areola complex, (b—arrow) hyperplastic glands and acinus, (c—arrow) areas of fibrosis and elastosis. Group (**D**): DMBA + RME 250 mg/kg treated animals showed (a—arrow) fibrosis with myxoid degeneration and individual cells of necrosis seen. Group (**E**): DMBA + RME 500 mg/kg showed (a—arrow) non-neoplastic glands and acinus, (b—arrow) fibrosis and occasional areas of necrosis seen. Group (**F**): VIDMBA + RME-EnCNPs 250 mg/kg treated animal shows (a—arrow) single layered non-neoplastic acinus. Group (**G**): DMBA + RME-EnCNPs 500 mg/kg treated animal shows (a—arrow) disappearance of tumor cells with no glands and acini noted, (b—arrow) fibrosis noted.

In Group VI (DMBA + RME-EnCNPs 250 mg/kg), a single-layered non-neoplastic acinus was observed. Finally, Group VII (DMBA + RME-EnCNPs 500 mg/kg) showed a disappearance of tumor cells, and fibrosis was reduced (Figure 12).

##### Histology of Liver of DMBA-Induced Mammary Tumor-Bearing Rats

**Figure 13:**
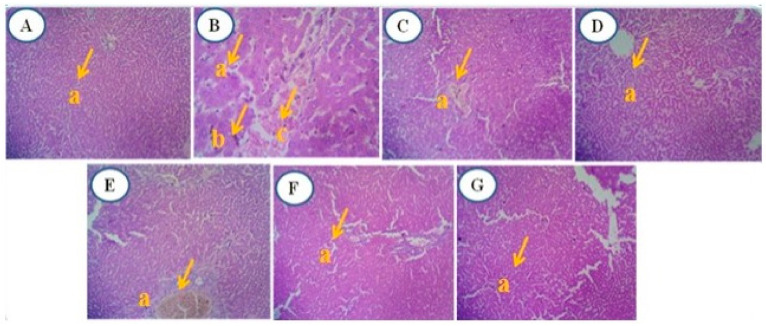
Histology of liver of DMBA-induced mammary tumor-bearing rats. (**A**–**G**) Shows histopathological analysis of liver tissue of control and experimental groups. Group (**A**): control showed normal architecture of hepatocytes (a—arrow) normal architecture. Group (**B**): DMBA-induced cancer bearing animal showed (a—arrow) abnormal architecture, (b—arrow) hepatocytes arranged in cords, (c—arrow) portal tract appears remarkable. Group (**C**): DMBA + standard (tamoxifen) treated animals showed (a—arrow) central veins with congestion. Group (**D**): DMBA + RME 250 mg/kg showed (a—arrow) sinusoidal dilatation. Group (**E**): DMBA + RME 500 mg/kg showed (a—arrow) normal hepatocytes with sinusoidal dilatation. Group (**F**): DMBA + RME-EnCNPs 250 mg/kg treated animals showed (a—arrow) normal hepatocytes with portal tract. Group (**G**): DMBA + RME-EnCNPs 500 mg/kg treated animals showed (a—arrow) normal hepatocytes with central vein congestion.

The histomorphology of liver tissues in the control and experimental groups was examined. Group I (control animals) showed the normal architecture of hepatocytes. In Group II (DMBA-treated animals), an abnormal architecture was observed, with hepatocytes arranged in cords, and the portal tract appeared remarkable. Group III (DMBA + standard—tamoxifen) exhibited central veins with congestion. Group IV (DMBA + RME 250 mg/kg) showed sinusoidal dilatation. Group V (DMBA + RME 500 mg/kg) displayed normal hepatocytes with a portal tract. In Group VI (DMBA + RME-EnCNPs 250 mg/kg) treated animals, normal hepatocytes with a portal tract were observed. Finally, Group VII (DMBA + RME-EnCNPs 500 mg/kg) showed normal hepatocytes with central vein congestion (Figure 13).

##### Histology of Kidney of DMBA-Induced Mammary Tumor-Bearing Rats

The histomorphology of kidney tissues in the control and experimental groups was examined. Group I (control animals) showed the normal architecture of kidney tissues. In Group II (DMBA-treated animals), glomeruli with loss of mesangial matrix expansion were observed. Group III (DMBA + standard—tamoxifen) displayed normal glomeruli.

**Figure 14:**
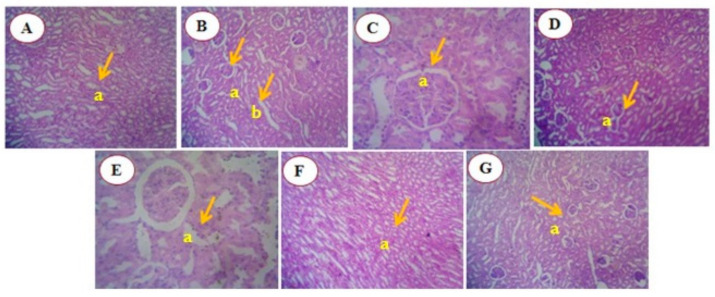
Histology of kidney of DMBA-induced mammary tumor-bearing rats. (**A**–**G**) Shows histopathological analysis of kidney tissue of control and experimental groups. Group (**A**): control showed (a—arrow) normal architecture. Group (**B**): DMBA-induced cancer bearing animals showed (a—arrow) glomeruli, (b—arrow) loss of mesangial matrix expansion. Group (**C**): DMBA + standard (tamoxifen) treated animals showed (a—arrow) normal glomeruli. Group (**D**): DMBA + RME 250 mg/kg showed (a—arrow) focal mild eosinophilic material in lumen. Group (**E**): DMBA + RME 500 mg/kg showed (a—arrow) no significant pathology in glomeruli. Interstitium showed normal. VI Group (**F**): DMBA + RME-EnCNPs 250 mg/kg treated animals showed (a—arrow) blood vessels with mild congestion. Group (**G**): DMBA + RME-EnCNPs 500 mg/kg treated animals showed (a—arrow) normal morphology in interstitium.

Group IV (DMBA + RME 250 mg/kg) showed focal mild eosinophilic material seen within the lumen. Group V (DMBA + RME 500 mg/kg) showed no significant pathology of glomeruli, and the interstitium appeared normal. In Group VI (DMBA + RME-EnCNPs 250 mg/kg) treated animals, normal blood vessels with mild congestion were observed. Finally, Group VII (DMBA + RME-EnCNPs 500 mg/kg) showed normal interstitium and normal morphology (Figure 14).

##### Histology of Uterus of DMBA-Induced Mammary Tumor-Bearing Rats

The histomorphology of uterus tissues in the control and experimental groups was examined. Group I (control animals) showed the normal architecture of uterus tissues. In Group II (DMBA-treated animals), abnormal epithelium with scattered inflammatory infiltrates was observed. Group III (DMBA + standard-tamoxifen) displayed glands with a circular and elongated shape.

**Figure 15:**
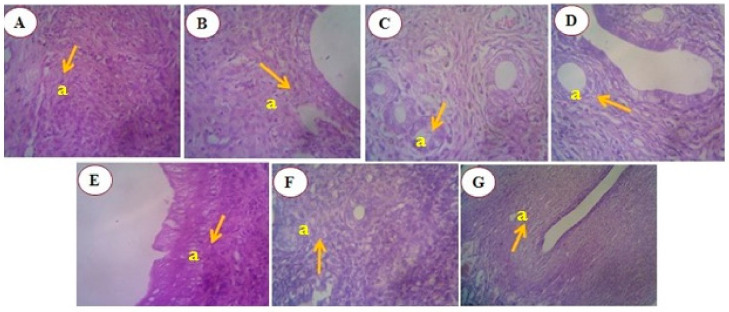
Histology of uterus of DMBA-induced mammary tumor-bearing rats. (**A**–**G**) Shows histopathological analysis of uterus tissue of control and experimental groups. Group (**A**): control showed (a—arrow) normal architecture. Group (**B**): DMBA-induced cancer bearing animals showed (a—arrow) abnormal epithelium with scattered inflammatory infiltrates. Group (**C**): DMBA + standard (tamoxifen) treated animals showed (a—arrow) glands with circular and elongated shape. Group (**D**): DMBA + RME 250 mg/kg showed (a—arrow) stroma with scattered inflammatory infiltrates. Group (**E**): DMBA + RME 500 mg/kg showed (a—arrow) normal mucosa. Group (**F**): DMBA + RME-EnCNPs 250 mg/kg treated animals showed (a—arrow) neutrophils and eosinophils. Group (**G**): DMBA + RME-EnCNPs 500 mg/kg treated animals showed (a—arrow) normal endomyometrium with scattered infiltrates.

Group IV (DMBA + RME 250 mg/kg) showed a stroma with scattered inflammatory infiltrates. Group V (DMBA + RME 500 mg/kg) showed normal mucosa. In Group VI (DMBA + RME-EnCNPs 250 mg/kg) treated animals, normal neutrophils and eosinophils were observed. Finally, Group VII (DMBA + RME-EnCNPs 500 mg/kg) showed normal endomyometrium with scattered infiltrates (Figure 15).

## 4. Discussion

The biodegradable polymer chitosan has been widely employed in drug delivery applications. Chitosan (C), derived from the N-deacetylation of chitin, a natural polysaccharide, is primarily present in the exoskeletons of crustaceans, insects, and fungi [42]. Cross-linking chitosan with sodium tripolyphosphate (TPP) represents a mild and efficient method to produce chitosan nanoparticles (CNPs). These particles are primarily formed through the electrostatic interaction between the positively charged chitosan and the negatively charged TPP molecules. Chitosan nanocarriers have demonstrated significant potential for various challenging drug delivery applications. The ionic gelation method used for preparing chitosan-TPP nanoparticles involves the spontaneous aggregation of chitosan with TPP, facilitated by the formation of molecular linkages between TPPs and chitosan amino groups [43].

Encapsulation plays a crucial role in protecting bioactive compounds from degradation. Currently, there are two types of encapsulations: microencapsulation and nanoencapsulation. Nanoencapsulation is preferred over microencapsulation due to its nanoscale size. Smaller particles not only enhance bioavailability but also allow for better control over their release [44]. Green chemistry strategies are favored for synthesizing nanomaterials replacing hazardous chemicals with eco-friendly biological materials during the synthesis of various therapeutic nanomaterials. This approach helps minimize the adverse impact of chemicals on human health [45].

In this study, the rise in drug entrapment efficiency with the escalation of plant extract concentration suggests that initially the nanoparticles were not saturated with the plant extract. The heightened concentration of the crude extract increased the drug’s availability for encapsulation. Additionally, the size of the nanoparticles plays a role in encapsulation efficiency, where larger nanoparticles, with their increased volume, can accommodate a greater quantity of the drug. In our investigation, the size of the root methanolic extract-encapsulated nanoparticles (RME-EnCNPs) was determined to be 83 nm. Similar findings were reported in [46].

### 4.1. Charaterization of RME-EnCNPs

The characterization of the RME-EnCNPs in this study involved various techniques, including UV–visible spectroscopy, FTIR, XRD, SEM, and zeta potential. The UV–vis absorption peak observed for the RME-EnCNPs in the range of 200–800 nm indicated the presence of CO groups in the CNPs. The FTIR peaks shifting from 3390.24 cm^−1^, 1025.94 cm^−1^, and 611.324 cm^−1^ for the CNPs to 3423.03 cm^−1^, 1095.37 cm^−1^, and 701.96 cm^−1^ for the RME-EnCNPs suggested the successful loading of the root methanolic extract of A. venenata into the CNPs. The sizes of the CNPs and RME-EnCNPs were found to be 79 nm and 83 nm, respectively, with the interaction between the polymer and plant extract leading to an increase in RME-EnCNPs compared to CNPs [47]. SEM analysis revealed almost separated spherical-shaped NPs for both CNPs and RME-EnCNPs, as is consistent with previous findings [48,49].

The zeta potential analysis revealed a higher value for the RME-EnCNPs (40.6 mV) compared to the CNPs (33.5 mV), indicating the excellent stability of the encapsulated drug. Moving on to the in vitro drug release and kinetic models, the release profile of RME-EnCNPs was examined, demonstrating that 50% of the drug was released within 2 h in a rapid and sustained manner. By the 5th hour, 87.8% of the drug had been released. This release pattern aligns with the two common profiles observed in drug release from nanoparticles: an initial burst phase due to drug adsorption onto the NP surface, followed by a sustained release attributed to slow drug diffusion from the polymer matrix and carrier matrix breakdown [50].

The observed initial burst release of the drug within the first two hours is likely due to factors such as the high surface area of the drug particles, rapid dissolution of the drug, and the nature of the formulation matrix, which may allow for faster release at the surface compared to the core [50]. This burst phase is common in drug delivery systems and can be influenced by the solubility and physicochemical properties of the drug, as well as the release mechanism of the carrier [51]. Addressing this burst release in future formulations may involve optimizing drug encapsulation techniques, adjusting the matrix composition, or exploring alternative carriers to achieve a more controlled, sustained release profile [52].

Drug release profiles can be influenced by mechanisms like simple diffusion, erosion, degradation, and absorption activity [51]. To design pharmaceutical formulations effectively, researchers have introduced various mathematical models. Drug carriers exhibit variations in shape, dimension, drug dissolution properties, and functionality. Due to these diversities, developing a universal model for predicting release profiles is not feasible. Hence, it becomes crucial to carefully select the most appropriate model for each specific research case [52].

A comprehensive kinetic analysis of RME release from chitosan nanoparticles was conducted using four mathematical models: zero order (cumulative percentage of drug release vs. time), first order (logarithmic cumulative percent drug remaining vs. time), Higuchi (cumulative percentage of drug release vs. square root of time), and Korsmeyer–Peppas (logarithmic cumulative percent drug release vs. log time) [53]. The zero order model indicated a gradual release of the drug, while the first order model described the absorption and/or elimination of the drug from the nanocarrier. The Higuchi model, which best fit the release profile with an R² value of 0.994, suggests a diffusion-controlled release mechanism. This indicates that RME is gradually released from the nanoparticles through diffusion, making it ideal for sustained drug delivery. Under physiological conditions, this diffusion-driven release ensures controlled drug release, maintaining therapeutic levels over time. Such sustained release is particularly beneficial for cancer treatment, where prolonged drug exposure is crucial for effective therapy and minimizing side effects. The Korsmeyer–Peppas model was used to assess the transport mechanisms involved in the release, whether by diffusion or swelling [54].

### 4.2. Acute Oral Toxicity Study

The outcomes of the acute toxicity analysis in the current study for both RME and RME-EnCNPs revealed no signs of toxicity or mortality in rats when administered orally at doses of 500, 1000, and 2000 mg/kg. Following the guidelines of the OECD (Organization for Economic Development, 2001) for the chemical labeling and classification of acute toxicity, RME and RME-EnCNPs would fall into category 5, considering their LD50 values greater than 2000–5000 mg/kg. This classification denotes the lowest toxicity class [55].

### 4.3. Assessment of Body Weight

DMBA, a known carcinogen, has been identified for its ability to induce mammary carcinoma in mice, particularly originating from the ductal elements of the mammary gland, by significantly increasing oxidative stress [56]. Throughout the 20-week study, a gradual increase in body weight was observed across all animal groups. Notably, a significant increase (*p* < 0.001) in body weight was observed in Group III (DMBA + STD) and Group VII (DMBA + RME-EnCNPs 500 mg/kg) during this period. Furthermore, rats administered RME at doses of 250 and 500 mg/kg exhibited a noteworthy increase (*p* < 0.05) in body weight at the end of the 20th week compared to Group II (DMBA 25 mg/kg). The injection of DMBA might have disrupted the body’s normal biochemical processes, affecting the functioning of antioxidant enzymes, leading to epithelial cell death, elevated lipid peroxidation, and increased levels of free radicals, collectively contributing to cellular membrane damage. Consequently, these factors could have influenced the overall body weight. The findings align with previous studies [57,58,59].

### 4.4. Tumor Weight, Volume, Incidence, and Burden

Sprague Dawley rats are commonly employed in DMBA-induced carcinogenesis experiments to induce mammary tumors, with DMBA recognized as an efficient inducer of mammary carcinoma after a single dose administration [60]. In the current study, a single dose of DMBA (25 mg/kg) was administered to 30-day-old Sprague Dawley rats. Tumor weight, volume, incidence, and burden were assessed at the end of the 20th week. A significant decrease (*p* < 0.001) in tumor weight, volume, incidence, and burden was observed in Group VII (DMBA + RME-EnCNPs 500 mg/kg) during this period compared to Group III (DMBA + STD tamoxifen). Standard control tamoxifen, a selective estrogen receptor modulator, inhibits the effects of the reproductive hormone estrogen, which plays a crucial role in the growth and development of various breast cancers [61,62].

Inflammation within the tumor microenvironment is associated with increased invasiveness and poor prognosis in various cancers, including breast cancer. Critical mediators of the inflammatory response include cytokines like interleukin-6 (IL-6), tumor necrosis factor-alpha (TNFα), and interleukin-1 beta (IL-1β). Numerous studies have linked these cytokines to breast cancer progression [63].

### 4.5. Biochemical Parameters

Given the vital roles of the liver and kidneys in the body, any disturbance in their normal functions can significantly impede various metabolic processes, leading to increased overall body toxicity. The liver, being the primary organ responsible for metabolizing xenobiotic substances, is susceptible to damage from chemical agents. In the present study, the carcinogenic metabolites and reactive oxygen species (ROS) resulting from DMBA metabolism were implicated in liver degradation [64].

The rise in total serum protein concentration observed may be attributed to changes in albumin and other proteins, collectively known as globulins. Alterations in serum albumin concentration are known to occur under conditions of oxidative stress, such as that associated with cancer [64]. The majority of serum proteins produced in the liver serve as biomarkers for liver dysfunction. In this investigation, significantly higher levels of total bilirubin, SGOT, SGPT, and ALP were noted in DMBA-treated animals compared to the control group. These elevated levels indicate DMBA-induced hepatic impairment [65]. SGOT and SGPT are enzymes involved in liver function, and their increase suggests impaired liver function, possibly due to tumor invasion [66]. ALP activity is vital for membrane permeability, molecule transport, protein synthesis, and glycogen metabolism. The significant reduction in these parameters after treatment with RME and RME-EnCNPs (250 and 500 mg/kg) indicates the potential hepatoprotective effect of A. venenata methanolic root extract. Flavonoids present in the extract may contribute to this hepatoprotective activity by aiding in the restoration of damaged liver portions [67,68].

Obesity and high total cholesterol have long been associated with an increased incidence of tumors in breast carcinoma animal models. Cholesterol, susceptible to oxidation, produces more lipid peroxidation products during oxidative stress. Hypercholesterolemia and oxidative stress are subsequent effects of rising cholesterol levels, leading to the activation of cells and the formation of oxygen free radicals [69]. In the present investigation, increased levels of total cholesterol and triglycerides were observed in the DMBA-induced rats (Group II) compared to Groups III to VII. Low HDL-cholesterol levels are often associated with higher estrogen levels in obese or overweight women, representing a significant risk factor for breast cancer [68]. Group II (DMBA- induced breast cancer rats) and Group VII (500 mg/kg of RME-EnCNPs treated) exhibited decreased and increased levels of HDL-cholesterol, respectively, as is consistent with prior investigations [69].

The kidneys, responsible for excreting harmful metabolic waste products, are unable to escape the detrimental effects of DMBA’s hazardous metabolic by-products. The present investigation noted significantly higher urea, uric acid, and creatinine levels in the DMBA-treated group compared to the control group, indicative of DMBA-induced renal impairment. However, after the administration of RME and RME-EnCNPs (250 and 500 mg/kg), a significant reduction in urea, uric acid, and creatinine levels was observed. This reduction in serum kidney biomarker levels suggests a renal protective effect of *A. venenata* root methanolic extract. The phenols and flavonoids present in this extract may contribute to the observed renal protective effect by aiding in the regeneration of damaged kidney components [70,71].

### 4.6. In Vivo Antioxidant Activity

In general, oxidative stress and the production of ROS have been connected to cancer progression. Compared to their normal cells, tumor cells show higher ROS levels and the tight regulation of REDOX homeostasis to maintain a low level of oxidative stress. Conventionally, several antioxidants have been extensively investigated to counteract breast carcinogenesis and tumor advancement as chemopreventive agents [72]. The total protein level in the rats administrated with DMBA was significantly decreased, in comparison with the rats treated with (200 and 500 mg/kg) RME and RME-EnCNPs. Similar results were reported in [73]. SODs use metal ions such as copper (Cu^2+^), zinc (Zn^2+^), manganese (Mn^2+^), or iron (Fe^2+^) as cofactors for catalyzing the dismutation of superoxide anion to oxygen and hydrogen peroxide. These metalloenzymes present in different compartments of the eukaryotic and prokaryotic cells and are extremely specific in regulating coupled biological processes [74]. Catalase (CAT) assists the decomposition of hydrogen peroxide to water and oxygen. It is chiefly located in cytosol and the peroxisomes of eukaryotes [75]. GPX is a cytosolic enzyme that catalyzes the breakdown of hydrogen peroxide and organic hydroperoxides [76]. Glutathione also known as GSH is present in the liver and protects cells from oxidative stress by reducing disulfide bonds of cytoplasmic proteins to cysteines [77]. Many studies have indicated that lipid peroxidation may be involved in tumor progression because this process can produce reactive and toxic metabolites. Malondialdehyde (MDA) is one of the important aldehydes of lipid peroxidation, which can react with proteins, DNA, and other biomolecules and modify their structure and function. In recent years, the determination of the MDA of tissues and plasma has been extensively followed in various cancer studies including breast cancer. Several research reports indicate the possibility of a relation between lipid peroxidation and breast cancer [78].

This study explores the therapeutic potential of *A. venenata* root methanolic extract (RME) for counteracting oxidative stress in tumor cells. In rats with DMBA-induced breast cancer, elevated oxidative stress was indicated by increased LPO and decreased levels of antioxidant enzymes. Treatment with RME-EnCNPs (500 mg/kg) significantly reversed these alterations, improving levels of total protein, SOD, catalase, GPx, and GSH, while reducing LPO (*p* < 0.001). The nanoparticles not only enhance the bioavailability and targeted delivery of RME’s bioactive compounds but also provide sustained antioxidant effects at the tumor site. By encapsulating RME in chitosan, a biopolymer with natural antioxidant properties, the nanoparticles facilitate continuous reactive oxygen species (ROS) scavenging, helping to neutralize oxidative damage [79,80,81]. This dual effect—enhanced drug delivery and ROS neutralization—reduced tumor growth and progression, highlighting the potential of RME-EnCNPs as a promising strategy for breast cancer treatment. Our findings supported the common observation that breast malignancies are connected to an increased level of SOD, catalase, GPx, and GSH and a decreased level of MDA [79,80,81].

### 4.7. Glycolytic and Non-Glycolytic Enzymes

Hexokinase, catalyzing the first step of glycolysis by phosphorylating glucose with ATP to produce glucose 6-phosphate, has four isoforms (HK1, HK2, HK3, and HK4) expressed in mammalian tissues. Cancer cells typically express high levels of HK2 compared to normal cells [82]. In the current study, DMBA-induced rats exhibited elevated levels of hexokinase, while RME-EnCNPs-treated animals showed reduced levels. Utilizing natural plant-derived secondary metabolites, which regulate glycolysis enzymes, holds promise for cancer treatment. For instance, physciosporin, a potent anticancer lichen compound, downregulates HK2 mRNA expression and inhibits breast cancer cell proliferation [83]. Similarly, morusin (flavonoid) and tanshinone IIA (diterpenoid) inhibit hepatocellular carcinoma and oral squamous cell carcinoma, respectively, with the suppression of HK2 enzyme [84,85].

Aldolase, a ubiquitous cytosolic enzyme in glycolysis, catalyzes the conversion of fructose 1-6-diphosphate to glyceraldehyde 3-phosphate and dihydroxyacetone phosphate. Classified into three groups (ALDOA, ALDOB, and ALDOC), ALDOA has been implicated in promoting tumor growth and metastasis in various cancers. The phosphorylation of ALDOA can enhance glucose metabolism in liver cancer cells, promoting their growth and tumor formation [86,87]. In the current study, aldolase levels were elevated in DMBA-induced rats and decreased in RME-EnCNPs-treated animals.

Glycogen has been linked to cancer cell survival, preventing glycogen breakdown, inducing apoptosis, and promoting early cell senescence in cancer cells. Glucose-6-phosphatase (encoded by G6PC) mediates the final step of gluconeogenesis, and its overexpression has been observed in glioblastomas. Fructose-1,6-bisphosphatase (FBPase), a key enzyme in gluconeogenesis, plays critical roles in tumor initiation and progression. Lower FBPase expression is associated with advanced tumor stages and worse prognoses in cancer patients [88,89]. In the present study, G6PC and FBPase levels were reduced in DMBA-induced rats and RME-EnCNPs-treated animals.

### 4.8. Estimation of Mitochondrial TCA Cycle Enzymes

Mitochondria are central to cellular functions, including ATP production, metabolite generation, and the regulation of various cellular processes. They communicate with the cell through mechanisms such as cytochrome c release, AMP-activated protein kinase activation, reactive oxygen species (ROS) generation, and mitochondrial DNA release. Recently, the release of TCA cycle metabolites from mitochondria has been shown to influence cell function, macromolecule biosynthesis, and epigenetic modifications [90].

Key enzymes in the TCA cycle, such as isocitrate dehydrogenase (IDH), succinate dehydrogenase (SDH), and malate dehydrogenase (MDH), play critical roles in cellular metabolism. Mutations in IDH1 and IDH2 are common in cancers like gliomas and acute myeloid leukemia [91]. SDH deficiency is linked to tumor progression, with succinate accumulation stabilizing HIF-1α. Increased SDHA expression has been associated with reduced cell proliferation in ovarian cancer cells [92]. MDH is involved in regenerating cytosolic NAD+, a process essential for cancer cell proliferation [93].

In this study, the activities of TCA cycle enzymes (IDH, SDH, and MDH) were significantly reduced in DMBA-induced breast cancer animals, likely due to alterations in cell morphology, mitochondrial structure, and reduced mitochondrial numbers. This decline may also be related to deficiencies in the electron transport chain. Treatment with RME-EnCNPs led to a significant increase in the activities of these enzymes, suggesting a protective effect of the encapsulated root methanolic extract. These findings align with previous reports by Senthilnathan et al. [94], who observed decreased enzyme activities in lung cancer-bearing animals.

### 4.9. Estimation of Hematological Parameters

Red blood cell count, hemoglobin levels, and packed cell volume are key markers for anemia, which in cancer patients is associated with an increased risk of cardiovascular events and mortality [95]. Cancer-related anemia can be caused by bleeding, nutritional deficiencies, bone marrow damage, tumor invasion, or the malignancy itself. Low mean corpuscular hemoglobin concentration (MCHC) indicates insufficient hemoglobin in red blood cells [96]. In this study, animals treated with 500 mg/kg RME-EnCNPs showed improved levels of these parameters, while the DMBA-induced mammary tumor-bearing animals exhibited decreased levels. Cell cannibalism, where cancer cells phagocytose their own or other cells (e.g., neutrophils and lymphocytes), is another hallmark of cancer [96]. Low white blood cell (WBC) counts increase infection risk, while elevated WBC counts are linked to invasive breast cancer development [97]. In this study, the DMBA-induced tumor-bearing animals had higher WBC counts and polymorphonuclear cell percentages compared to those treated with RME-EnCNPs. These findings align with previous studies, showing significant decreases in hemoglobin and red blood cell indices, along with increased platelet and WBC counts in the DMBA-induced breast cancer-bearing animals compared to Ficus carica latex-treated groups [98].

### 4.10. Histological Analysis

The histopathological analysis of mammary tissues from the cancer-bearing animals revealed malignant epithelial cells with hyperchromasia, increased nuclear/cytoplasmic ratio, and pleomorphism. In contrast, animals treated with 500 mg/kg RME-EnCNPs showed the disappearance of tumor cells, with no glandular or acinar structures and areas of marked fibrosis. Examination of liver tissues in the cancer-bearing animals revealed abnormal architecture, with hepatocytes arranged in cords and prominent portal tracts, likely due to carcinogen-induced free radical damage to DNA [99]. In the RME-EnCNPs-treated animals, these changes were resolved, possibly due to the antioxidant properties of the extract. The treated animals exhibited normal hepatocytes with central vein congestion, suggesting liver protection.

Kidney tissues from the cancer-bearing animals showed glomerular damage and tubular abnormalities, while the RME-EnCNPs-treated animals exhibited normal interstitial morphology, indicating preserved kidney function and detoxification processes [100]. The examination of uterine tissues in the cancer-bearing animals revealed abnormal epithelium and scattered inflammatory infiltrates. In contrast, the RME-EnCNPs-treated animals showed a normal endomyometrium with mild infiltrates.

### 4.11. Mechanism of Action and Molecular Pathways

The anticancer effects of RME-EnCNPs are likely driven by multiple mechanisms, primarily involving the generation of reactive oxygen species (ROS), which induces oxidative stress and leads to apoptosis in cancer cells. The bioactive compounds in *Alstonia venenata*, including alkaloids and flavonoids, are released from the chitosan nanoparticles in a sustained manner, enhancing the drug’s stability, bioavailability, and therapeutic impact. These compounds may also modulate key signaling pathways, such as PI3K/Akt and MAPK/ERK, which are critical for cell survival and proliferation, contributing to tumor growth inhibition. Additionally, mitochondrial dysfunction, indicated by changes in TCA cycle enzyme activities, may further drive the anticancer effects of RME-EnCNPs.

Nanoparticle-based drug delivery systems have gained significant attention in cancer therapy due to their ability to enhance the stability, bioavailability, and targeted delivery of therapeutic agents. Studies by Yang et al. [101] and Zhang et al. [102] have demonstrated that chitosan nanoparticles loaded with anticancer drugs, such as doxorubicin and paclitaxel, significantly improve drug uptake and tumor targeting, resulting in reduced tumor growth and metastasis. These studies suggest that the anticancer effects of nanoparticle-loaded drugs are mediated by molecular mechanisms like ROS generation and apoptosis induction. Additionally, research on plant extracts, including *Alstonia venenata*, supports the notion that natural compounds can target multiple signaling pathways involved in cancer progression. Malik et al. [12] demonstrated that the ethanolic extract of *Alstonia venenata* leaves exhibits anticancer activity. In vitro studies showed cytotoxic effects on DLA, EAC, and normal splenocytes. The tumor-bearing animals treated with 100 mg/kg, 250 mg/kg, and 500 mg/kg doses of the extract had significant increases in survival, with the 500 mg dose extending lifespan by 30.79%. The results also indicated tumor growth inhibition, as reflected in changes in body weight, consistent with the findings in our study, where RME-EnCNPs effectively inhibited tumor growth and improved antioxidant activity [10,11,12].

Our results further align with studies on herbal extract-loaded nanoparticles, such as Patil et al. [101], who reported enhanced bioavailability and sustained drug release using chitosan nanoparticles. The diffusion-controlled release mechanism observed for RME-EnCNPs, as described by the Higuchi model, highlights the potential of chitosan-based systems for controlled, prolonged therapeutic effects. The anticancer activity of RME-EnCNPs likely involves a combination of ROS-induced oxidative stress, mitochondrial dysfunction, and the modulation of key signaling pathways like PI3K/Akt and MAPK/ERK. These findings underscore the advantages of the nanoparticle-mediated delivery of herbal extracts over conventional drug formulations, offering improved therapeutic outcomes with targeted delivery and reduced toxicity.

## 5. Conclusions

In this study, we evaluated the chemoprotective potential of RME-loaded chitosan nanoparticles (RME-EnCNPs) against DMBA-induced mammary gland tumors. Treatment with RME-EnCNPs demonstrated a dose-dependent effect, significantly reducing tumor markers, biochemical parameters, antioxidant levels, enzymatic activity, hematological parameters, and histopathological abnormalities. These results suggest that RME-EnCNPs have strong potential for preventing breast cancer.

Furthermore, the study highlights that RME-EnCNPs are more effective than free RME, providing enhanced tumor inhibition and sustained drug release. This positions RME-EnCNPs as a promising, less toxic alternative to conventional chemotherapy. However, to fully realize their clinical potential, further research is required, including toxicity assessments, long-term release studies, and an in-depth exploration of their mechanisms of action. Advanced preclinical models will also be crucial for evaluating the clinical feasibility of RME-EnCNPs.

## Figures and Tables

**Table 1:** Correlation coefficients of different kinetics models for root methanolic extract-encapsulated chitosan nanoparticles (RME-EnCNPs).

Kinetic Models	Encapsulated Chitosan Nanoparticles		
	R^2^	Slope	Intercept
Zero order kinetics	0.966	17.47	24.77
First order kinetics	0.942	0.398	16.47
Higuchi model	0.994	50.306	44.57
Korsmeyer–Peppas model	0.908	8.105	32.10

**Table 2:** Effect of root methanolic extract (RME) and root methanolic extract-encapsulated chitosan nanoparticles (RME-EnCNPs) on body weight of female Sprague Dawley rats.

Treatment	Initial Treatment of Body Weight (g) (4 Weeks Old)	Final Treatment of Body Weight (g) (20 Weeks Old)
Group I (Control)	125 ± 0.36	215 ± 1.3
Group II (DMBA 25 mg/kg)	131 ± 0.42	135 ± 1.0
Group III (DMBA + STD 10 mg/kg)	138 ± 0.46	190 ± 1.6 ***
Group IV (DMBA + RME 250 mg/kg)	130 ± 0.53	153 ± 1.3 **
Group V (DMBA + RME 500 mg/kg)	140 ± 0.56	166 ± 1.8 **
Group VI (DMBA + RME-EnCNPs 250 mg/kg)	142 ± 0.6	175 ± 1.7 **
Group VII (DMBA + RME-EnCNPs 500 mg/kg)	139 ± 0.59	185 ± 1.9 ***

Results expressed by triplicate means (*n* = 3) ± Standard error of mean (SEM). Statistical significance determined with Dunnett’s t-distribution with probability value, *** *p* < 0.001, ** *p* < 0.01, calculated by comparing treated group with control group. DMBA-7, 12-Dimethylbenz[a]anthracene; STD, standard (tamoxifen); RME, root methanolic extract; RME-EnCNPs, root methanolic extract-encapsulated chitosan nanoparticles.

**Table 3:** Effect of root methanolic extract (RME) and root methanolic extract-encapsulated chitosan nanoparticles (RME-EnCNPs) on tumor weight, volume, incidence, and burden of female Sprague Dawley rats.

Group	Group I	Group II Only	Group III	Group IV	Group V	Group VI	Group VII
	Control	DMBA-	DMBA + STD	DMBA + RME	DMBA + RME	DMBA + RME-	DMBA + RME-
		25 mg/kg	10 mg/kg	250 mg/kg	500 mg/kg	EnCNPs 250 mg/kg	EnCNPs 500 mg/kg
Tumor Weight (mg/g)	----	20 ± 12.2 *	3.44 ± 0.59 ***	15.2 ± 3.96 **	8.04 ± 2.9 *	6.7 ± 2.9 **	4.42 ± 0.878 ***
Tumor Volume (cm^3^)	----	5.3 ± 0.054 *	1.1 ± 0.045 ***	4.5 ± 0.169 *	3.3 ± 0.092 *	2.3 ± 0.049 **	1.3 ± 0.024 ***
Tumor Burden (g)	----	9.67 ± 0.882 *	2.33 ± 0.882 ***	7.33 ± 0.882 *	5.4 ± 0.882 **	3.2 ± 0.577 **	2.84 ± 0.577 ***
Tumor Incidence (%)	----	8.33 ± 0.333 *	2.17 ± 0.333 ***	6.67 ± 0.882 **	4.6 ±0.577 *	3.83 ± 0.882 **	2.21 ± 0.333 ***

Results are expressed by triplicate means (*n* = 3) ± Standard error of mean (SEM). In statistical significance determined with Dunnett’s test-distribution with probability value, *** *p* < 0.001, ** *p* < 0.01, * *p* < 0.05 calculated by comparing DMBA-treated group with RME and RME-En CNPs-treated group. DMBA-7,12-Dimethyl benz[a]anthracene; STD, standard (tamoxifen); RME, root methanolic extract; RME-EnCNPs, root methanolic-extract-encapsulated chitosan nanoparticles.

**Table 4:** Effect of root methanolic extract (RME) and root methanolic extract-encapsulated chitosan nanoparticles (RME-EnCNPs) on bilirubin, serum glutamic oxaloacetic transaminase (SGOT), serum glutamate pyruvate transaminase (SGPT), alkaline phosphatase (ALP) total estrogen, progesterone, total cholesterol, triglycerides, high density level (HDL)-cholesterol, total urea, uric acid, and creatinine levels in female Sprague Dawley rats.

Group	Group I (Control)	Group II (Only DMBA 25 mg/kg)	Group III (DMBA + STD 10 mg/kg)	Group IV (DMBA + R ME 250 mg/kg)	Group V (DMBA + R ME 500 mg/kg)	Group VI (DMBA + R ME-EnCNPs (250 mg/kg)	Group VII (DMBA + R ME-EnCNPs (500 mg/kg)
Total Bilirubin (mg/dL),	2.35 ± 0.263	39.2 ± 0.346	2.15 ± 0.144 ***	30.1 ± 0.404 **	24.1 ± 0.231 **	12.6 ± 0.173 **	1.3 ± 0.289 ***
SGOT (u/L)	35 ± 2.89	139 ± 1.73	38 ± 1.73 ***	92 ± 2.31 **	75 ± 4.04 **	62.5 ± 0.866 **	40 ± 4.04 ***
SGPT (u/L)	33.3 ± 2.6	107 ± 5.2	34 ± 1.0 ***	84 ± 6.35 **	68.3 ± 2.6 **	57.7 ± 6.06 **	36.7 ± 0.82 ***
ALP (u/L)	125 ± 4.04	302 ± 31.2	128 ± 2.31 ***	190 ± 0.882 **	179 ± 5.77 **	155 ± 13.6 **	130 ± 4.62 ***
Est.H (Pg/mL)	62.8 ± 18.5	98 ± 4.7	58.6 ± 13.6 ***	89.9 ± 1.25 ***	75.2 ± 1.325 **	64.4 ± 1.28 **	50.1 ± 1.98 ***
Prog.H (ng/mL)	44.52 ± 0.36	92.52 ± 0.95	46.3 ± 1.63 ***	84.2 ± 0.652 **	72.9 ± 0.12 **	60.5 ± 0.165 **	43.9 ± 0.39 ***
Total Cholesterol (mg/dL)	148 ± 1.44	215 ± 8.6	150 ± 5.77 ***	195 ± 37.5 **	186 ± 5.77 **	175 ± 14.4 *	151 ± 2.89 ***
Triglycerides (mg/dL)	95.3 ± 1.47	183 ± 3.7	96.2 ± 1.36 ***	179 ± 3.41 **	158 ± 0.202 *	122 ± 3.72 *	98 ± 2.34 ***
HDL-Cholesterol (mg/dL)	68.8 ± 1.67	22.9 ± 3.49	63.8 ± 0.635 ***	33.9 ± 5.17 **	46.1 ± 0.751 *	52.6 ± 2.14 **	65.2 ± 0.318 ***
Urea (mg/dL)	19.8 ± 1.67	76 ± 4.07	20.1 ± 3.18 ***	66.9 ± 2.34 **	55.3 ± 0.866 *	40.7 ± 3.41 *	20.7 ± 0.52 ***
Uric acid (mg/dL)	3.05 ± 0.26	44.2 ± 0.34	2.8 ± 0.231 ***	38.05 ± 0.43 **	26.1 ± 0.316 *	14.05 ± 0.144 *	2.3 ± 0.231 ***
Creatinine (mg/dL)	0.55 ± 0.144	28.3 ± 0.52	1.2 ± 0.23 ***	18.5 ± 0.202 **	11.8 ± 0.15 *	8.2 ± 0.346 *	1.6 ± 0.404 ***

Results expressed by triplicate means (*n* = 3) ± standard error of mean (SEM). Statistical significance determined with Dunnett’s t-distribution with probability value, *** *p* < 0.001, ** *p* < 0.01, * *p* < 0.05 calculated by comparing treated group with control group. DMBA-7, 12-Dimethylbenz[a]anthracene; STD, standard (tamoxifen); RME, root methanolic extract; RME-EnCNPS, root methanolic extract-encapsulated chitosan nanoparticles.

**Table 5:** Effect of root methanolic extract (RME) and root methanolic extract-encapsulated chitosan nanoparticles (RME-EnCNPs) on total protein, superoxide dismutase activity (SOD), catalase activity, glutathione peroxidase (GPX), glutathione content (GSH), and lipid peroxidation (LPO) levels in female Sprague Dawley rats.

Group	Group I Control	Group II Only DMBA25 mg/kg	Group III DMBA + STD	Group IV DMBA + RMEL.D	Group V DMBA + RMEH.D	Group VI DMBA + RME-	Group VII DMBA + RME-EnCNPs
			10 mg/kg	250 mg/kg	500 mg/kg	EnCNPsL.D	H.D500 mg/kg
						250 mg/kg	
Total Protein (mg/dL)	25.2 ± 0.09	8.1 ± 0.06	24.8 ± 0.05 ***	13.5 ± 0.08 **	15.7 ± 0.06 **	18.2± 0.07 *	23.3 ± 0.02 ***
SOD (Unit/min/mg protein)	9.27 ± 0.09	0.32 ± 0.06	10.25 ± 0.02 ***	3.282 ± 0.08 **	4.233 ± 0.07 **	6.21 ± 0.05 *	11.25 ± 0.06 ***
CATALASE (Unit /min/mg protein)	12.3 ± 0.04	1.01 ± 0.07	11.57 ± 0.04 ***	3.672 ± 0.08 **	8.529 ± 0.01 **	10.57 ± 0.04 **	16.51 ± 0.04 ***
GPX (nmol of Glutathione peroxidase/min/mg protein)	36.3 ± 0.82	5.02 ± 0.46	32.03 ± 0.26 ***	10.08 ± 0.15 **	15.53 ± 0.02 **	24.03 ± 0.03 *	35.1 ± 0.15 ***
GSH (nmol of Glutathione hyroxidase/min/mg protein)	37.35 ± 0.04	0.36 ± 0.04	35.09 ± 0.03 ***	5.76 ± 0.05 **	10.24 ± 0.02 **	25.41 ± 0.04 *	36.18 ± 0.08 ***
LPO (nmol of MDA/mg protein)	3.54 ± 0.03	23.41 ± 0.04	4.29 ± 0.73 ***	19.54 ± 0.93 **	14.24 ± 0.01 **	11.79 ± 0.01 *	5.26 ± 0.01 ***

Results expressed by triplicate means (*n* = 3) ± standard error of mean (SEM). Statistical significance determined with Dunnett’s test-distribution with probability value, *** *p* < 0.001, ** *p* < 0.01, * *p* < 0.05 calculated by comparing treated group with control group. DMBA-7, 12-Dimethylbenz[a]anthracene; STD, standard (tamoxifen); RME, root methanolic extract; RME EnCNPs, root methanolic extract-encapsulated chitosan nanoparticles.

**Table 6:** Effect of root methanolic extract (RME) and root methanolic extract-encapsulated chitosan nanoparticles (RME-EnCNPs) and hematological parameters in rats with DMBA–induced mammary tumors.

Group I	Group II Only DMBA	Group III DMBA + STD	Group IV DMBA + RMEL.D	Group V DMBA + RME	Group VI DMBA + RME-EnCNPs	Group VII DMBA + RME-EnCNPs
Group Control	25 mg/kg	10 mg/kg	250 mg/kg	500 mg/kg	250 mg/kg	500 mg/kg
RBC (×10^6^/µL) 15.01 ± 0.10	1.12 ± 0.139	17.38 ± 0.237 ***	5.92 ± 0.397 *	8.57 ± 0.32 *	12.6 ± 0.283 **	16.07 ± 0.109 ***
WBC (×10^3^/µL) 16.1 ± 0.896	61 ± 1.29	18.4 ± 0.503 ***	45.6 ± 0.867 *	37.9 ± 0.551 *	28.4 ± 1.38 *	17.3 ± 0.689 ***
HB (g/dL) 15.7 ± 0.669	1.9 ± 0.491	13.7 ± 1.06 ***	2.6 ± 0.788 *	4.6 ± 1.1 *	10.3 ± 0.29 **	14.3 ± 0.265 ***
PCV (%) 40.3 ± 1.92	3.8 ± 1.23	36.1 ± 3.34 ***	5.9 ± 2.28 *	18.3 ± 3.18 *	28.3 ± 1.48 *	37.3 ± 0.882 ***
Polymorphs (%) 12.7 ± 0.667	52 ± 1.53	13.2 ± 1.15 ***	47.3 ± 1.45 *	32.3 ± 0.882 *	21.3 ± 0.88 *	15.6 ± 1.45 ***
Lymphocytes (%) 1.3 ± 2.67	52.7 ± 3.28	87.3 ± 0.882 ***	51.3 ± 0.333 **	68.7 ± 0.667 *	79.3 ± 1.2 **	88 ± 3.21 ***
Monocytes (%) 59.33 ± 1.33	6.67 ± 1.45	58.7 ± 0.882 ***	26.3 ± 1.2 *	35.7 ± 0.333 *	42.7 ± 0.33 **	54.3 ± 1.45 ***
Eosinophils (%) 39.67 ± 0.66	6.67 ± 0.333	37 ± 0.577 ***	16.2 ± 0.577 *	24.4 ± 0.333 *	29.7 ± 0.33 *	38.7 ± 0.882 ***
MCH (Pg) 35.8 ± 1.15	8.4 ± 1.07	34.8 ± 0.767 ***	11.3 ± 0.794 *	18.5 ± 0.841 *	26 ± 0.695 *	32.6 ± 0.99 ***

Results expressed by triplicate means (*n* = 3) ± standard error of mean (SEM). Statistical significance determined with Dunnett’s t-distribution with probability value, *** *p* < 0.001, ** *p* < 0.01, * *p* < 0.05 calculated by comparing treated group with control group. DMBA-7, 12-Dimethylbenz[a]anthracene; STD, standard (tamoxifen); RME, root methanolic extract; RME-EnCNPS, root methanolic extract-encapsulated chitosan nanoparticles.

## Data Availability

The raw data supporting the conclusions of this article will be made available by the authors on request.
